# Impaired Basal Forebrain Cholinergic Neuron GDNF Signaling Contributes to Perioperative Sleep Deprivation–Induced Chronicity of Postsurgical Pain in Mice Through Regulating Cholinergic Neuronal Activity, Apoptosis, and Autophagy

**DOI:** 10.1111/cns.70147

**Published:** 2024-12-05

**Authors:** Dong Wang, Shi‐Nan Wei, Lu Zhang, Zhi‐Chen Lang, Si‐Nian Wang, Bo Cheng, Yan Lu, Xiu Wang, Wei Wang, Feng‐Sheng Li, Hao Zhang

**Affiliations:** ^1^ The Postgraduate Training Base of Jinzhou Medical University and Department of Anesthesiology The PLA Rocket Force Characteristic Medical Center Beijing China; ^2^ Department of Anesthesiology Tangdu Hospital, Air Force Military Medical University Xian Shanxi China; ^3^ Department of Anesthesiology Zibo Central Hospital Zibo China; ^4^ Department of Nuclear Radiation Injury and Monitoring The PLA Rocket Force Characteristic Medical Center Beijing China; ^5^ Department of Pathology The PLA Rocket Force Characteristic Medical Center Beijing China; ^6^ Department of Neurology The PLA Rocket Force Characteristic Medical Center Beijing China; ^7^ Department of Pediatrics Zhengzhou Central Hospital Zhengzhou China

**Keywords:** apoptosis, basal forebrain cholinergic neuron, chronic postsurgical pain, glial‐derived neurotrophic factor (GDNF), sleep deprivation

## Abstract

**Aims:**

This study investigated the roles of lateral basal forebrain glial cell line–derived neurotrophic factor (GDNF) signaling and cholinergic neuron activity, apoptosis, and autophagy dysfunction in sleep deprivation–induced increased risk of chronic postsurgical pain (CPSP) in mice.

**Methods:**

Sleep deprivation (6 h per day from −1 to 3 days postoperatively) was administered to mice receiving skin/muscle incision and retraction (SMIR) to determine whether perioperative sleep deprivation induces mechanical and thermal pain hypersensitivity, increases the risk of chronic pain, and causes changes of basal forebrain neurons activity (c‐Fos immunostaining), apoptosis (cleaved Caspase‐3 expression), autophagy (LC3 and p62 expression) and GDNF expression. Adeno‐associated virus (AAV)‐GDNF was microinjected into the basal forebrain to see whether increased GDNF expression could reverse sleep deprivation–induced changes in pain duration and cholinergic neuron apoptosis and autophagy. Cholinergic neurons were further depleted by mu p75‐SAP to examine whether the pain‐prolonging effects of sleep deprivation still exist.

**Results:**

Perioperative sleep deprivation enhanced pain sensation and prolonged pain duration in SMIR mice, which was accompanied by decreased cholinergic neuron activity and GDNF expression, increased apoptosis, and autophagy dysfunction in the substantia innominata (SI), magnocellular preoptic nucleus (MCPO), and horizontal diagonal band Broca (HDB) (hereafter lateral basal forebrain). Normalizing cholinergic neuron GDNF expression by AAV‐GDNF in the lateral basal forebrain inhibited apoptosis and autophagy dysfunction and mitigated sleep deprivation–induced pain maintenance. Mice with selective lesion of lateral basal forebrain cholinergic neurons were resistant to the pain‐enhancing and prolonging effects of sleep deprivation and the pain‐alleviating effects of AAV‐GDNF therapy.

**Conclusions:**

Perioperative sleep deprivation promotes chronicity of postsurgical pain possibly through decreasing basal forebrain GDNF signaling and causing cholinergic neuronal apoptosis and autophagy dysfunction.

## Graphical abstract

Surgery combined with perioperative sleep deprivation induces decreased GDNF in the lateral basal forebrain, which leads to enhanced apoptosis and autophagy dysfunction of cholinergic neurons and increased risk of chronic postsurgical pain. Normalizing GDNF levels by AAV‐GDNF therapy rescues the deleterious effects of perioperative sleep deprivation. Selective lesion of the cholinergic neurons of lateral basal forebrain by mu p75‐SAP decreases the pain threshold and abolishes the pain‐enhancing effects of sleep deprivation.

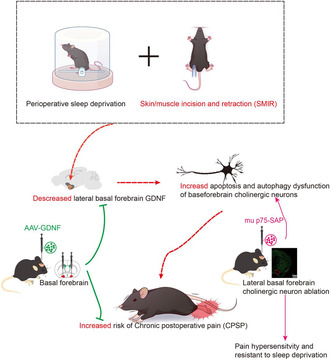

## 1 Introduction

Chronic postoperative pain (CPSP) is a persistent state of pain after surgery that often complicates postoperative recovery, causes adverse physical and psychological trauma for patients, and increases healthcare costs [1, 2]. More than 300 million surgeries are performed worldwide each year [3], and the prevalence of CPSP is approximately 10% after all surgical procedures [4]. There is a strong need to elucidate the risk factors and corresponding mechanisms of CPSP.

Pre‐ and peri‐operative sleep disturbances are highly prevalent among surgical patients [5, 6, 7, 8]. Emerging animal and clinical studies have found a probable association between sleep deficiency and the development of CPSP [9, 10, 11, 12]. Our previous study found that perioperative sleep deprivation administered to mice with skin/muscle incision and retraction (SMIR), a model that mimics pain following major or invasive surgeries, activates spinal inflammation, thereby inducing an increased risk of chronic postoperative pain [13]. However, the supraspinal mechanisms involved in the promotion of CPSP following sleep deprivation remain unclear.

Sleep deprivation exerts a profound influence on a multitude of brain signaling pathways and neural circuits, which may be implicated in the pathogenesis of chronic pain [14]. Notably, the basal forebrain, which includes the medial septum/diagonal band complex (MS/DB), the ventral pallidum (VP), the substantia innominata (SI), the nucleus basalis (NBM), magnocellular preoptic nucleus (MCPO), and so on, is enriched in cholinergic neurons but also with glutamatergic neurons [15, 16] and essential for chronic pain modulation [17, 18, 19]. Choline acetyltransferase (ChAT), a protein responsible for synthesis of acetylcholine, is a specific functional marker for cholinergic neurons [20]. Vesicular glutamate transporter 2 (VGluT2) transports glutamate into synaptic vesicles before release and is a widely recognized marker for glutamatergic neurons [21]. A recent study found that during the nonrapid eye movement (NREM) sleep phase, mice with peripheral nerve injury exhibited increased activation of ChAT‐positive cholinergic neurons in the anterior nucleus basalis of the basal forebrain. These activated neurons release more acetylcholine (Ach) to the primary somatosensory cortex and activate vasoactive intestinal polypeptide‐expressing interneurons there, leading to disinhibition of local pain‐inhibitory circuits and mechanical allodynia in mice [17]. Nevertheless, Oswald et al. [19] found that c‐Fos expression, an indicator for neuronal activation, was increased in the NBM in a mouse model of inflammatory and neuropathic pain. Optogenetic stimulation of cholinergic neurons in the NBM area increases Ach release in the prelimbic cortex (PL), leading to the activation of layer 5 pyramidal neurons, which facilitated the activation of the downstream nociceptive modulation system, thereby relieving abnormal mechanical pain in mice. Moreover, excitatory glutamatergic transmission in the MS sustains the chronic neuropathic pain in the rodent chronic constriction injury (CCI) model [22]. Besides neuronal activity and neurotransmitter release, the degeneration of basal forebrain cholinergic neurons might also modulate pain maintenance. The rodent cholinergic basal forebrain neurons express the p75 neurotrophin receptor (p75^NTR^), whose sustained activation induced neuronal apoptosis, and can be lesioned selectively by the immunotoxin 192 IgG‐saporin (SAP) directed against the rat p75^NTR^ or mu p75‐SAP targeting the mouse p75^NTR^ [23, 24]. Lesion of the basal forebrain cholinergic neurons with 192 IgG‐saporin in rats reduces operant escape from cold and heat stimulation when tested over 15–19 weeks after injection in rats [25]. However, there are currently no relevant studies on the relationship between basal forebrain cholinergic neuron signaling and sleep deprivation–induced chronicity of postoperative pain, which differs a lot from chronic neuropathic pain in mechanisms [26, 27].

There are multiple forms of neuronal degeneration caused by sleep deprivation such as apoptosis and autophagy [22, 28]. Cleaved Caspase‐3 is a reliable marker for apoptotic cells [29]. The levels of endogenous LC3II/I and p62 serve to monitor autophagic flux [30]. Moreover, GDNF, a neurotrophin with wide distribution [31, 32], was microinjected into the lateral ventricles of the rats and rabbits [33]. In addition, researchers have demonstrated that GDNF pays a contribution to pain regulation [34, 35] and is a crucial molecule in reducing apoptosis of neurons and regulating cognitive function [34, 36, 37]. Based on the evidence, the present study was designed to test the hypothesis that basal forebrain cholinergic neuronal activity, apoptosis and autophagy dysfunction, and GDNF signaling may regulate sleep deprivation–induced chronicity of postoperative pain.

## 2 Materials and Methods

### 2.1 Animals

To rule out the gender‐dependent differences in pain sensation and maintenance [38, 39] and reduce the number of mice sacrificed, the study only used 6 to 8‐week‐old male mice of C57BL/6J strains, weighing around 20 g on average, which were bought from Beijing SPF Biotechnology Co. Ltd., (China). They were allowed to acclimate for a minimum of 1 week prior to any experimental procedures. Mice were housed in cages with unlimited access to food and water, maintained at a temperature range of 20°C–25°C, and subjected to a 12:12‐h light–dark cycle (light on at 08:00). The experimental protocols were designed under the guidance of the ethical society and adhered to the guidelines outlined in the Guide for the Care and Use of Laboratory Animals (8th Edition, The National Academies Press, Washington DC, USA, 2011) and the International Pain Research Association's Policy on the Use of Laboratory Animals. Based on our previous sample size calculations [13], which considered a hypothesis predicting chronic pain incidences (defined as *a* ≥ 50% drop in mechanical pain threshold at day 21 post‐SMIR compared with baseline) of 0.5 for SMIR mice and 0.9 for SMIR mice subjected to additional sleep deprivation, each group comprised 10 mice.

### 2.2 Study Design

**FIGURE 1:**
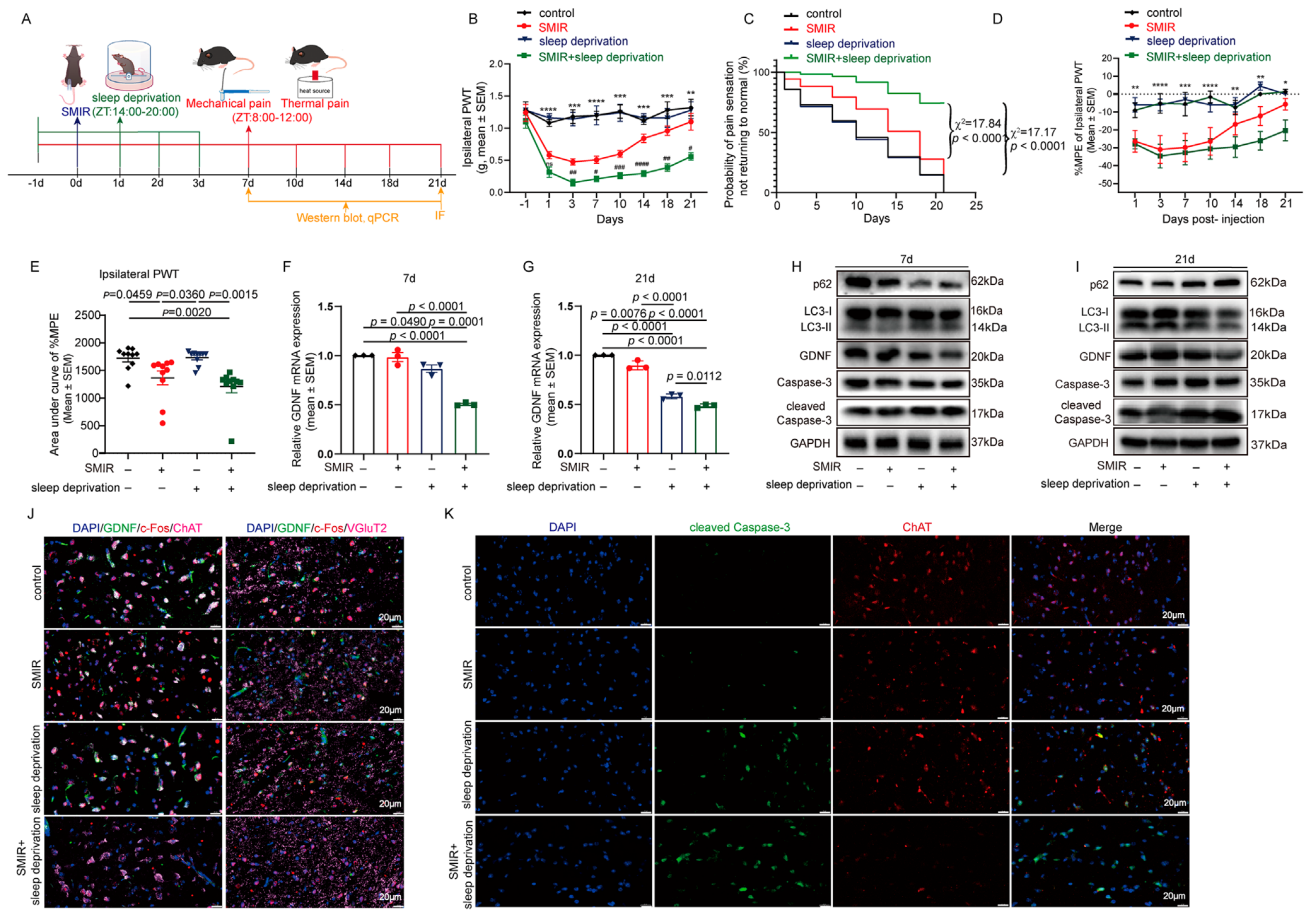
Perioperative sleep deprivation promotes chronicity of postsurgical pain with decreased GDNF contents and increased cholinergic neuronal apoptosis and autophagy dysfunction in the basal forebrain. (A) Study design. A mouse SMIR model was established, which received 6 h of total sleep deprivation daily from 1 day prior to until 3 days after the surgery, and mechanic and heat‐evoked pain was measured until 21 days after surgery. (B, C) Perioperative sleep deprivation was found to significantly increase mechanical pain intensity (B) and prolong pain maintenance (C) in SMIR mice. (D, E) Percentage of maximal possible effect (%MPE) of groups at different time points (D) and area under the curve (AUC) of %MPE (E). (F, G) Expression of GDNF mRNA at 7 (F) and 21 (G) days after surgery. (H, I) GDNF contents, and markers of apoptosis (Caspase‐3 and cleaved Caspase‐3) and autophagy (LC3 and p62) expression across groups at 7 (H) and 21 (I) days after surgery. (J) Multiplex immunofluorescence (IF) results showing an increased staining of cleaved Caspase‐3 in ChAT‐positive cholinergic neurons. (K) Immunofluorescence showed decreased expression of GDNF in both ChAT‐positive cholinergic neurons and VGluT2‐positive glutamatergic neurons, and decreased c‐Fos expression in ChAT‐positive cholinergic neurons only. Ten mice per group were used for the behavioral tests. Biochemical experiments were performed in three independent samples. *p < 0.05; **p < 0.01; ***p < 0.001; and ****p < 0.0001, SMIR + sleep deprivation versus control. ^#^ *p* < 0.05; ^##^ *p* < 0.01; ^###^ *p* < 0.001; and ^####^ *p* < 0.0001, SMIR + sleep deprivation versus SMIR. ChAT, choline acetyltransferase; SEM, standard error of the mean; SMIR, skin/muscle incision and retraction; VGluT2, vesicular glutamate transporter 2.

**FIGURE 2:**
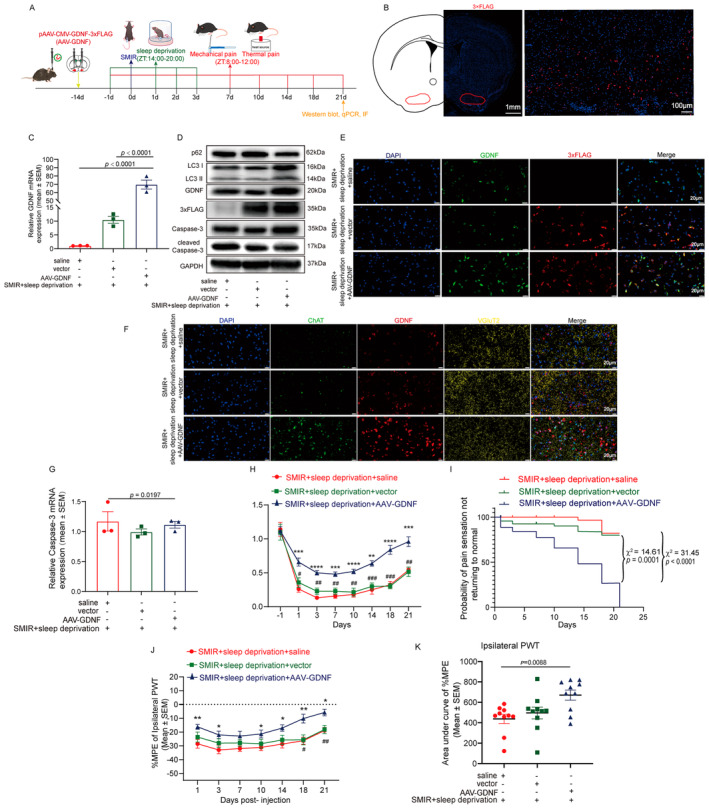
AAV‐GDNF promotes GDNF expression, reduces cholinergic neuronal apoptosis and autophagy dysfunction, and counteracts sleep deprivation‐induced postoperative chronic pain. (A) Study design. Mice were injected bilaterally into the lateral basal forebrain with AAV‐GDNF, vector, or saline control. A mouse SMIR model was established, which received 6 h of total sleep deprivation daily from 1 day prior to until 3 days after the surgery, and mechanic and heat‐evoked pain was measured until 21 days after surgery. (B) Immunofluorescence staining showing strong 3 × FLAG expression in the lateral basal forebrain, indicating accurate microinjection and successful overexpression. (C) Expression of GDNF mRNA at 21 days after surgery. (D) GDNF contents, 3 × FLAG Tag, and markers of apoptosis (cleaved Caspase‐3 and Caspase‐3) and autophagy (LC3 and p62) expression across groups at 21 days after surgery. (E) Immunofluorescence showing increased expression of GDNF in mice receiving AAV‐GDNF intervention. (F) Multiplex immunofluorescence showing increased GDNF expression by AAV‐GDNF in both ChAT‐positive cholinergic neurons and VGluT2‐positive glutamatergic neurons. (G) Expression of Caspase‐3 mRNA at 21 days after surgery. (H, I) AAV‐GDNF counteracts surgery plus sleep deprivation‐induced mechanical allodynia (H) and prolonged pain maintenance (I). (J, K) %MPE at different time points (J) and AUC of %MPE (K). Ten mice per group were used for the behavioral tests. Biochemical experiments were performed in three independent samples. *p < 0.05; **p < 0.01; ***p < 0.001; and ****p < 0.0001, SMIR + sleep deprivation + saline versus SMIR + sleep deprivation + AAV‐GDNF. ^#^ *p* < 0.05; ^##^ *p* < 0.01; ^###^ *p* < 0.001; and ^####^ *p* < 0.0001, SMIR + sleep deprivation + vector versus SMIR + sleep deprivation + AAV‐GDNF. AAV‐GDNF, AAV2/9 carrying pAAV‐CMV‐Gdnf‐3×FLAG‐P2A‐mCherry‐WPRE; ChAT, choline acetyltransferase; VGluT2, vesicular glutamate transporter 2.

**FIGURE 3:**
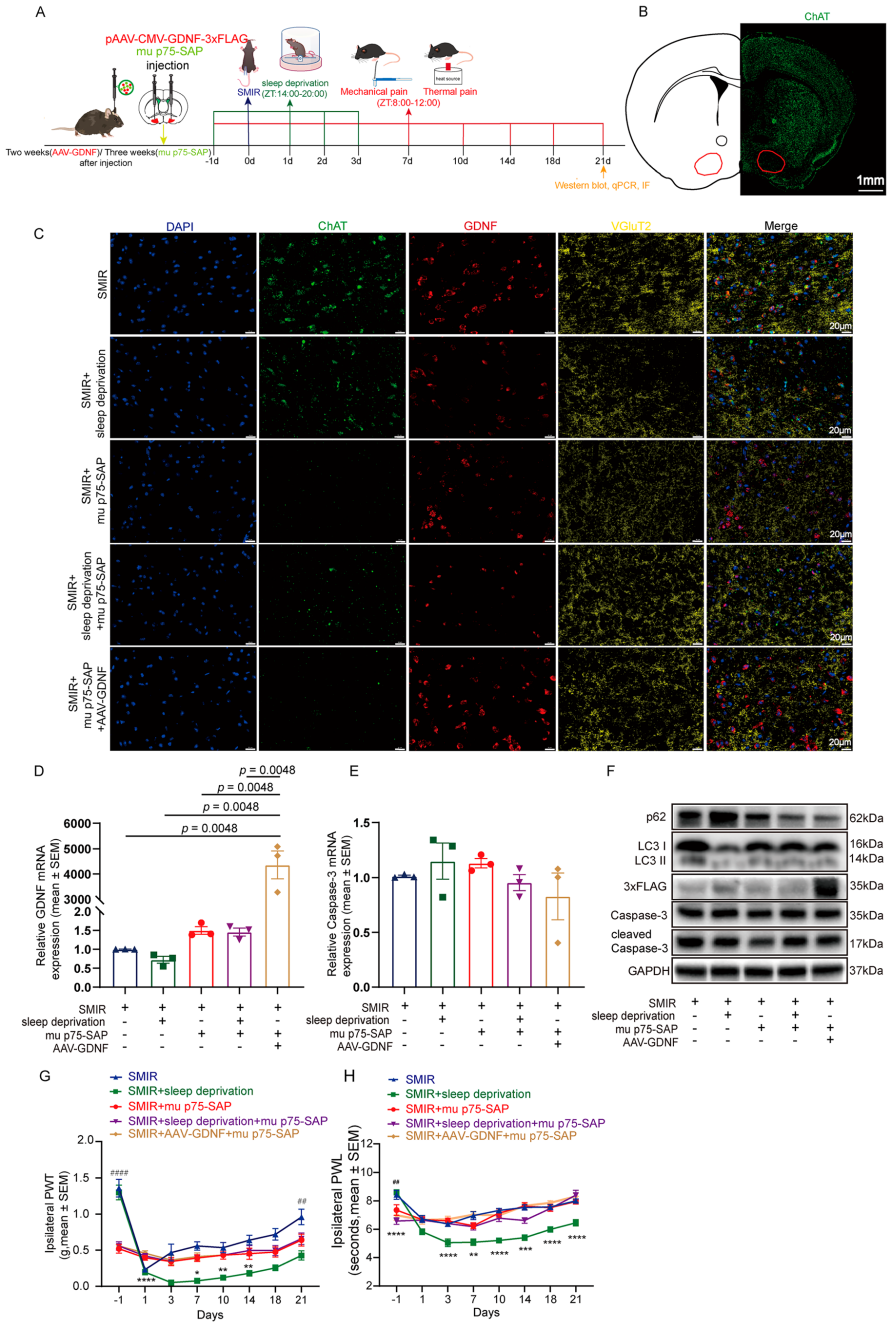
Mice with lesions of lateral basal forebrain cholinergic neurons are resistant to the pain‐enhancing effects of sleep deprivation and the pain‐alleviating effects of AAV‐GDNF therapy. (A) Study design. Mice were injected bilaterally in the lateral basal forebrain with AAV‐GDNF or mu p75‐SAP before surgery. Mechanic and heat‐evoked pain was measured until 21 days after surgery. (B) Multiplex immunofluorescence showing substantially decreased immunostaining of ChAT‐positive cholinergic neurons after mu p75‐SAP treatment, which was not rescued by AAV‐GDNF treatment. (C) Immunofluorescence showing lesion of the lateral basal forebrain (substantia innominata (SI), magnocellular preoptic nucleus (MCPO), and the horizontal diagonal band Broca (HDB)) cholinergic neurons by mu p75‐SAP. (D, E) Expression of GDNF and Caspase‐3 mRNA at 21 days after surgery. (F) 3 × FLAG Tag, and markers of apoptosis (cleaved Caspase‐3 and Caspase‐3) and autophagy (LC3 and p62) expression across groups at 21 days after surgery. (G, H) Decreased mechanical (G) and thermal (H) pain threshold in mu p75‐SAP‐treated mice, which were not affected by further sleep deprivation or AAV‐GDNF treatment. Ten mice per group for the behavioral tests were used. Western blot and RT‐qPCR experiments were performed in three independent samples. *p < 0.05; **p < 0.01; ***p < 0.001; and ****p < 0.0001, SMIR + sleep deprivation + mu p75‐SAP versus SMIR + sleep deprivation. ^#^ *p* < 0.05; ^##^ *p* < 0.01; ^###^ *p* < 0.001; and ^####^ *p* < 0.0001, SMIR versus SMIR + mu p75‐SAP.

This study consisted of three experiments. First, the concomitant changes in basal forebrain neuronal activity (c‐Fos immunostaining), apoptosis (Caspase‐3 and cleaved Caspase‐3 measurement), autophagy (LC3 and p62 determination), and GDNF expression in SMIR mice subjected to perioperative sleep deprivation were examined (Figure 1A). Second, the ameliorating effects of normalizing GDNF expression in the lateral basal forebrain on sleep deprivation–induced prolonged pain hypersensitivity and basal forebrain neuron apoptosis were tested (Figure 2A). Third, lateral basal forebrain cholinergic neurons were targeted for ablation using mu p75‐SAP to observe the changes in pain sensitivity both at baseline and following sleep deprivation as well as the protective effects of normalized GDNF expression (Figure 3A).

### 2.3 Skin/Muscle Incision and Retraction (SMIR) Modeling

The SMIR model was established following previously outlined procedures by us [13]. In brief, the hair on the right hind limb was removed, and the saphenous vein was identified. A skin incision of 1.5–2 cm was made approximately 3 mm medial to the saphenous vein to expose the thigh muscle. Subsequent separation of superficial muscles facilitated the insertion of a miniature anatomical retractor, which retracted the skin and superficial muscles of the thigh by 1 cm, with retractor fascia underneath exposed. This retraction was maintained for 1 h before the closure of the skin and muscle. Mice in the control group underwent the same incision and exposure procedure but without retraction. Following recovery from anesthesia, animals displaying normal mobility, including the ability to walk and stand on their hind paws to access food and water, were selected for further experiments.

### 2.4 Stereotactic Surgery and Microinjection

Mice were positioned prone in a stereotaxic apparatus (YuYan, China) under anesthesia maintained with sevoflurane (4%–6%) in pure oxygen. Penicillin potassium was injected subcutaneously to prevent infection. To minimize incisional pain, ropivacaine (0.3%, 1 mL, AstraZeneca) was injected to infiltrate the local tissue. Following successful exposure of the dura, a 5‐μL microinjector with a minimum scale of 0.1 μL (33G, 15 mm; Hamilton, USA), containing either saline or virus at various concentrations, was bilaterally inserted into the lateral basal forebrain position (coordinates: anterior–posterior (AP) −0.01 mm, mediallateral (ML) ± 1.5 mm, and dorsalventral (DV) −5.0 mm, calculated in accordance with the mouse brain atlas by Paxinos and Franklin [40]). To ablate the basal forebrain cholinergic neurons, 0.4 μg/μL of mu p75‐SAP in 0.6 μL phosphate‐buffered saline (Cat. #IT‐16, Advanced Targeting Systems, USA) [41] was used 3 weeks before the SMIR modeling. To increase GDNF expression, AAV2/9 carrying pAAV‐CMV‐GDNF‐3×FLAG‐P2A‐mCherry‐WPRE (AAV‐GDNF) or its control (vector) was constructed by OBiO (China) and 0.25 μL (titer: 1.8 * 10^13^ vg/mL determined by PCR) of virus per side was microinjected slowly into the lateral basal forebrain 2 weeks before the SMIR modeling. The injection sites were verified by immunofluorescence staining. Relevant behavioral data were discarded if immunofluorescence showed inaccurate microinjections. New mice were added to ensure that the number of experimental animals per group reached 10.

### 2.5 Sleep Deprivation

An apparatus (XR‐XS108) with biofeedback mode developed by Xinruan Soft (China) was used for total sleep deprivation [13, 22]. The efficacy of this system for sleep deprivation has been previously confirmed [13]. Briefly, the system monitored the activities of the animals, and a sleep score with a range from 0 to 100 was calculated based on their behaviors. Zero indicates full wakefulness and activity, while 100 indicates complete sleep. When the score dropped to below 10, an interfering rod started rotating to wake the mice at a speed of 10 circles per minute for 1 min. Total sleep deprivation began at 08:00 and lasted for 6 h, once daily, beginning the day before surgery and continuing for 3 days postsurgery. Before sleep deprivation, mice were acclimated to the apparatus for 1 h. Throughout the sleep deprivation period, mice had unrestricted access to food and water. Control mice were allowed to move freely in the cylinder without any intervention.

### 2.6 Pain Evaluation

Animals were habituated and tested for baseline pain before the SMIR and sleep deprivation procedures. The assessment of mechanical pain was conducted using the von Frey test [42]. The mice were placed on a wire mesh in a separate box to acclimate for 30 min. The von Frey filaments (Stoelting, USA) were gently applied to the hind paw plantar surface in ascending order until they bent. The response was deemed positive if the mouse exhibited rapid retraction or licking of the paw within 5 s of filament application. A 30‐s interval was given between each trial, and the identical procedure was replicated for the contralateral hind paw. Stimulation was applied only when the mouse was standing on all four paws and not engaged in other activities such as walking, grooming, or sniffing. The paw withdrawal threshold (PWT) was determined as the minimum force necessary to evoke a positive response in at least three out of five tests.

The Hargreaves paw withdrawal latency (PWL) method was utilized for the evaluation of thermal nociceptive sensitivity [43]. Mice were individually placed in Plexiglas boxes with a glass floor. To test thermal pain perception, a focused laser light beam was directed onto the plantar surface of the hind paw. The intensity of the laser was adjusted carefully to a practical and safe level. Once the stimulation began, the timer started. The maximum exposure time was set at 20 s to avoid tissue damage. Upon withdrawal of the paw from the heat source, the timer stopped, and the withdrawal latency was recorded in seconds. Three measurements were taken for each hind paw, with alternating stimulation, and the average was calculated as the PWL. To avoid sensitization of the paw, a 10‐min interval was given between repeated tests.

The percentage of maximal possible effect (% MPE) was calculated according to the formulas below: For PWT, %MPE = (post‐SMIR PWT − baseline PWT)/ (cut‐off PWT (4 g)‐baseline PWT). For PWL, %MPE = (post‐SMIR PWL − baseline PWL)/ (cut‐off PWL (20 s)‐baseline PWL). The area under curve (AUC) was calculated as the accumulated %MPE during the observatory period obtained by the trapezoidal rule [44, 45].

### 2.7 Western Blot

Mouse lateral basal forebrain tissue was collected at 7 or 21 days after SMIR surgery and lysed with tissue protein lysates (YC365453; Thermo Fisher, USA) supplemented with a commercial kit of protease and phosphatase inhibitors (P002; NCM biotech, China). The lysates were then centrifuged at 13,000 RPM for 15 min at 4°C. The protein concentration in the supernatant was determined with the bicinchoninic acid (BCA) protein assay method (P0011; Beyotime, China). The proteins were separated using a 10% separating gel, and a polyvinylidene fluoride (PVDF) membrane was used for wet transfer. After blocking, primary antibodies including rabbit anti‐GDNF (bs23586R, 1:1000; Bioss, China), rabbit anti‐LC3B (ab192890, 1:1000; Abcam, UK), and rabbit anti‐p62 (ab109012, 1:1000; Abcam), rabbit anti‐Caspase‐3 (9665P, 1:1000; Cell Signaling Technology (CST), USA), rabbit anti‐cleaved Caspase‐3 (96,613, 1:1000; CST), mouse anti‐3 × FLAG tag (orb357990, 1:1000; Biorbyt, UK), and mouse anti‐GAPDH (60,004‐1‐IG, 1:1000; Proteintech, China) were used to incubate the membranes at 4°C overnight. After washing with TBST, the membranes were incubated with HRP‐conjugated secondary antibodies (goat anti‐rabbit and goat anti‐mouse, both 1:5000, ZRA03 and ZM03, YTHX Biotechnology) for an hour at room temperature. Bands were detected using the ChemiDoc XRS^+^ system (Bio‐Rad), and their intensities, with GAPDH as a control, were measured with Image J software (National Institutes of Health, USA).

### 2.8 Real‐Time Quantitative Polymerase Chain Reaction (RT‐qPCR)

RNA extracted from basal forebrain tissue by TRIzol reagent (93289; Sigma‐Aldrich, USA) was reverse transcribed into cDNA using the PrimeScript RT Master Mix kit with gDNA Eraser (RR036A; Takara, Japan). Real‐time PCR was conducted with the SYBR Green Taq Mix (RR820A; Takara) kit on the Bio‐Rad CFX Real‐Time PCR System with an annealing temperature of 60°C. Results were evaluated using the $2^{-\Delta \Delta C_{T}}$ method with GAPDH as a reference. The primers used were: p62 Forward: AGGATGGGGGACTTGGGTTGC, Reverse: TCACAGATCACATTGGGGGTGC; Caspase‐3 Forward: TGGGGACTGATGAGGAGA, Reverse: ACTGGATGAACCA CGAC; GDNF Forward: CAGTGACTCCAATATGCCTGA, Reverse: CCGCTTGT TTATCTGGTGAC; GAPDH Forward: GCCATCACTGCCACTCAGAA, Reverse: GGCATGTCAGATCCACAACG.

### 2.9 Immunofluorescence and Multiplex Immunofluorescence

Mice were euthanized 21 days post‐SMIR and perfused with cold phosphate‐buffered saline (PBS) followed by 4% paraformaldehyde (PFA). Brains were isolated, fixed in PFA, and then dehydrated in sucrose gradients. Coronal sections (10 μm) were prepared with a LEICA cryostat after tissue embedding. Antigen retrieval was done on basal forebrain sections in citrate buffer (pH = 6.0) using an autoclave, followed by goat serum (SL038; Solarbio, China) blocking.

For traditional immunofluorescence, mixed primary antibodies from distinct sources were applied overnight at 4°C. Afterward, sections were incubated with matching secondary antibodies for an hour at room temperature, away from light. Slides were then washed in PBS, stained with DAPI (G1012 for immediate use; Servicebio, China) for 10 min, and treated for autofluorescence (G1221; Servicebio, China) if required. The slides were then sealed with an antifade medium (G1901; Servicebio, China) before digitization via PANNORAMIC scanners (3DHISTECH, Hungry). Image analysis was completed with ImageJ software.

For multiplex immunofluorescence staining, after incubation with the primary antibody and washing, corresponding horseradish peroxidase (HRP)‐linked secondary antibodies and TSA (Tyramide signal amplification) fluorophore kits (G1231, G1232 and G1233, 1:500; Servicebio, China) were administered sequentially for serial staining.

The primary and secondary antibodies used for immunofluorescence were as follows: anti‐c‐FOS (DE,226008, 1:1000; synaptic systems), anticleaved‐Caspase‐3 (GB11532, 1:1000; Servicebio, China), anti‐GDNF (ab108319, 1:500; Abcam, USA), anti‐VGluT2 (GB11821‐100, 1:500; Servicebio, China), anti‐ChAT (GB11070‐100, 1:500; Servicebio); anti‐3 × FLAG (orb357990, 1:500; Biorbyt, UK); HRP‐conjugated goat antirabbit IgG (H + L) (GB23303, 1:500; Servicebio), and HRP‐conjugated goat antimouse IgG (H + L) (GB23301, 1:500; Servicebio). The images were scanned using a Pannoramic Desk Scanner (3DHistech), and viewed and cropped using CaseViewer software (version 2.3).

### 2.10 Statistical Analysis

GraphPad Prism 8.0 (USA) and SPSS Statistics version 27.0 (IBM, USA) were used for statistical analysis and graph plotting. The Shapiro–Wilk test was used for assessing measurement data normality. The PWT, PWL, PCR, and Western blot data in this study followed a normal distribution and were expressed as means ± standard errors of the means (SEMs). Differences among groups were evaluated using one‐way analysis of variance (ANOVA) with Tukey's post hoc test. Repeated measurement ANOVA (two‐way for grouping and time, or three‐way adding sleep deprivation) with post hoc Bonferroni's correction was used for the analysis of the PWT and PWL data, which were measured at multiple time points. For comparing abnormal mechanical pain duration (≥ 50% drop of PWT from baseline), the Kaplan–Meier survival curve was generated and the Breslow test was utilized. Statistical differences were considered to be present if the *p*‐value was smaller than 0.05 (two‐sided) or 0.025 (one‐sided).

## 3 Results

### 3.1 Perioperative Sleep Deprivation Promotes Postoperative Pain Chronicity With Decreased GDNF Contents and Increased Cholinergic Neuronal Apoptosis and Autophagy Dysfunction in the Lateral Basal Forebrain

The three‐way ANOVA showed significant interactions among time, SMIR, and sleep deprivation (*F* [1.368, 12.309] = 7.453, *p* = 0.013, Greenhouse & Geisser correction), a notable SMIR–sleep deprivation interaction (*F* [1, 9] = 29.321, *p* < 0.001), and a main effect of sleep deprivation (*F* [1, 9] = 14.504, *p* = 0.004, Figure 1B) for mechanical pain at the ipsilateral hind paws. Compared with the mice without SMIR or sleep deprivation, sleep‐deprived mice showed no changes in pain sensation. Compared with the SMIR mice, mice subjected to SMIR plus sleep deprivation showed lower mechanical pain threshold (Figure 1B) and smaller %MPE (Figure 1C) at 3–21 days after surgery, and smaller AUC of %MPE (Figure 1D). Analysis of the thermal pain revealed similar results (Figure S1A–C). The combined SMIR and sleep deprivation extended the duration of mechanical pain significantly compared to SMIR alone (*p* < 0.001, median 21 vs. 18 days, Figure 1E). Contralateral hind paw pain was unaffected by SMIR and/or sleep deprivation (Figure S1D,E).

Compared with the SMIR mice, SMIR mice with perioperative sleep disruption showed reduced GDNF mRNA and protein levels (Figure 1F–I, Figure S1F,G), and increased cleaved Caspase‐3/Caspase‐3 protein ratio (Figure 1H,I, Figure S1H,I), with no significant difference on Caspase‐3 mRNA levels (Figure 1J,K) at 7 and 21 days after surgery. As for the expression changes of autophagy markers, sleep deprivation decreases LC3 II/I ratio at 7 days after surgery compared with control and SMIR alone, with no significant difference at 21 days after surgery (Figure 1H,I, Figure S1L,M). The mRNA levels of p62 were not changed across groups at each time point (Figure S1N,O). No significant differences were found for p62 protein levels in SMIR mice receiving sleep deprivation or not (Figure 1H,I, Figure S1P,Q).

c‐Fos immunofluorescence indicated reduced neuronal activity in ChAT^+^ cholinergic neurons of the lateral basal forebrain in SMIR mice with sleep deprivation versus those with SMIR alone, with no change in VGluT2^+^ glutamatergic neurons (Figure 1J). The combined SMIR and sleep deprivation resulted in a reduction in GDNF protein content in both glutamatergic and cholinergic neurons when compared with SMIR alone (Figure 1J). The decrease in GDNF expression due to SMIR and sleep deprivation was associated with lowered ChAT activity and heightened cleaved Caspase‐3 levels in cholinergic neurons, suggesting enhanced apoptosis of cholinergic neurons compared with SMIR alone (Figure 1K).

### 3.2 AAV‐GDNF Promotes GDNF Expression, Reduces Cholinergic Neuronal Apoptosis and Autophagy Dysfunction, and Counteracts Sleep Deprivation–Induced Postoperative Chronic Pain

AAV‐GDNF microinjection into the lateral basal forebrain (Figure 2B) induced robust GDNF mRNA (Figure 2C) and protein expression (Figure 2D, Figure S2A) compared with saline and vector control in SMIR mice with sleep deprivation. The increased GDNF expression was confirmed by immunofluorescence (Figure 2E). Further multiplex immunofluorescence staining showed that increased GDNF was found in both ChAT‐positive cholinergic neurons and VGluT2‐positive glutamatergic neurons in the basal forebrain (Figure 2F).

When GDNF expression was increased by AAV‐GDNF injection in SMIR mice receiving sleep deprivation, the cleaved Caspase‐3/Caspase‐3 protein ratio (Figure 2D, Figure S2B) was decreased, with decreased Caspase‐3 mRNA levels (Figure 2G) at 21 days after surgery, indicating decreased apoptosis. Correspondingly, there was an increased number of ChAT‐positive cholinergic neurons after AAV‐GDNF therapy (Figure 2F). In addition, the ratio of LC3 II/I was increased (Figure 2D, Figure S2C), accompanied by decreased p62 protein contents (Figure 2D, Figure S2D) and no significant changes of p62 mRNA (Figure S2E) in the mice receiving AAV‐GDNF gene therapy compared with saline and vector control, indicating enhanced autophagy.

In behavioral tests, mice with AAV‐GDNF treatment exhibited lower pain intensity and larger %MPE (Figure 2J) at different time points, and larger AUC (Figure 2K) in mechanical (Figure 2H) and thermal (Figure S2F–H) pain tests and shorter median mechanical pain duration (Figure 2I) at the ipsilateral hind paws compared with those with saline and vector control. AAV‐GDNF administration had no substantial impact on the contralateral hind paw pain thresholds (Figure S2I,J).

### 3.3 Mice With Lesion of Lateral Basal Forebrain Cholinergic Neurons Are Resistant to the Pain‐Enhancing Effects of Sleep Deprivation and the Pain‐Alleviating Effects of AAV‐GDNF Therapy

Multiplex immunofluorescence staining revealed that ChAT‐positive signals were scattered and weak in the lateral basal forebrain of mice after mu p75‐SAP injection, while the signal of VGluT2 was intensified (Figure 3B,C) compared with those mice receiving vehicle injection, suggesting a successful lesion of lateral basal forebrain cholinergic neurons. AAV‐GDNF treatment increased GDNF mRNA and protein expression in the basal forebrain (Figure 3D,E) compared with saline control in mu p75‐SAP‐treated mice. mu p75‐SAP did not alter cleaved Caspase‐3/Caspase‐3 ratio, LC3 II/I ratio, p62 protein expression (Figure 3D, Figure S3A–C), or Caspase‐3 (Figure 3F) and p62 (Figure S3D) mRNA levels in SMIR mice. After mu p75‐SAP microinjection, further sleep deprivation or AAV‐GDNF treatment did not result in additional effects on the expression of markers of autophagy and apoptosis in SMIR mice (Figure 3D–F, Figure S3A–D).

Behavioral test results showed that mu p75‐SAP‐treated mice had reduced mechanical and thermal pain thresholds and smaller %MPE (Figure 2H) throughout the observational period, and smaller AUC (Figure 2I) at both the ipsilateral (Figure 3G–I and Figure S3E–G) and contralateral (Figure S3H,I) hind paws throughout the observational period. After mu p75‐SAP microinjection, the pain sensation in SMIR mice was not statistically affected by further sleep deprivation or AAV‐GDNF treatment (Figure 3G–I, Figure S3E–I).

## 4 Discussion

Our study found that surgery in conjunction with perioperative sleep deprivation induced a reduction in the content of GDNF in the lateral basal forebrain, a decrease in cholinergic neuronal activity, and an increase in the rates of cholinergic neuronal apoptosis and autophagy dysfunction, which was accompanied by increased pain sensitivity and the development of chronic postsurgical pain (Figure 1). Enhancing GDNF expression in the basal forebrain cholinergic neurons rescued the apoptosis and autophagy dysfunction of these neurons and the pain‐enhancing and prolonging effects caused by sleep deprivation combined with surgery (Figure 2). Lesion of the lateral basal forebrain cholinergic neurons using mu p75‐SAP made the mice more sensitive to pain stimulation, but resistant to the pain‐enhancing and prolonging effects of sleep deprivation (Figure 3).

The experiments revealed that both 6 h of sleep deprivation per day for 3 days and SMIR alone induced minimal decrease in GDNF mRNA and protein levels in the lateral basal forebrain while a synergistic effect was observed by PCR, Western blot, and multiple immunofluorescence results (Figure 1). The reducing effects of single surgery or sleep deprivation alone on brain GDNF levels were consistent with previous findings. Lin et al. [46] reported that after left tibial fracture surgery, the aged mice showed a significant decrease in GDNF levels in the hippocampus. Blood GDNF mRNA levels decreased in individuals subjected to 24 h of sleep deprivation [47]. In contrast, hippocampal tissue GDNF concentrations did not exhibit a significant change in rats that underwent a 48‐h period of postsurgical sleep deprivation following hernia repair compared with surgical rats without sleep deprivation [48]. The difference might be due to the differences in timing and brain areas harvested for GDNF detection, sleep deprivation strategies, and surgical treatments. Previous reports may give us some enlightenment on the molecular and cellular mechanisms underneath the synergistic effects of sleep deprivation and surgery on inhibiting GDNF expression. For example, GDNF promoter contains the nuclear factor‐kappa B (NF‐κB)‐responsive elements [49], which can be regulated by both surgery‐induced neuroinflammation [50] and sleep deprivation [51]. An in‐depth study of this might yield interesting results.

The modulating roles of central nervous system GDNF signaling in chronic pain have been verified by studies. In the context of spinal cord compression (SCC)‐induced chronic pain, the rat models with SCC demonstrated a reduction in the expression of GDNF mRNA and protein in the spinal cord tissues involved. Overexpression of GDNF inhibited neuron apoptosis and alleviated the nerve injury and pain caused by SCC [52]. GDNF expression was decreased in the lumbar spinal cord and dorsal root ganglion in a rat model of tibial bone cancer pain, but not in the anterior cingulate cortex. Lentiviral vector–mediated increase of GDNF expression in spinal cord reduced mechanical and thermal hyperalgesia [53]. When GDNF was injected into the locus coeruleus (LC) of rats with chronic sciatic nerve constriction injury, prolonged analgesic effects on mechanical allodynia and thermal hyperalgesia were observed [54]. Consistent with these evidences, in conjunction with alterations in GDNF expression within the lateral basal forebrain, we observed that sleep deprivation resulted in a notable increase in pain sensitivity and a significant prolongation of postoperative pain (Figure 1). Further GDNF overexpression results showed the increase of the lateral basal forebrain GDNF levels increased the mechanical and thermal pain threshold and shortened the duration of pain (Figure 2). These results supported the role of basal forebrain GDNF signaling in the development of chronic postsurgical pain. However, neuropathic pain and chronic postoperative pain have both similar and distinct pathogenesis and key targets [26, 27]. The specific molecular and cellular biological mechanisms of GDNF in both types of chronic pain remain to be elucidated.

We further examined the pain‐alleviating mechanisms of GDNF in the lateral basal forebrain. GDNF has been demonstrated to be beneficial for the survival and activation of a multitude of neurons, including basal forebrain cholinergic neurons [32, 52, 55, 56]. GDNF counteracts cholinergic neuronal damage caused by aging and axonal disconnection [32, 56] and maintains the survival of basal forebrain cholinergic neurons in vitro [56] and ex vivo [57]. Interestingly, Alzheimer's disease (AD), a condition with pathological loss of basal forebrain cholinergic function and significantly decreased serum GDNF levels [58], is also associated with a high burden of chronic pain [59]. In accordance with the aforementioned findings, we herein found c‐Fos expression, a marker of neuronal activation which can be induced by GDNF [60], was decreased in cholinergic neurons but not in glutamatergic neurons in SMIR mice with sleep. In the meantime, apoptosis of cholinergic neurons, as supported by increased cleaved Caspase‐3 expression was observed and autophagy was disrupted after surgery combined with sleep deprivation (Figure 1). Normalizing GDNF expression with AAV‐GDNF therapy in lateral basal forebrain cholinergic neurons significantly decreased Caspase‐3 expression and increased LC3II/I ratio in the basal forebrain, and shortened the duration of mechanical and thermal hyperalgesia induced by sleep deprivation in SMIR mice (Figure 2). Furthermore, when cholinergic neurons were depleted by mu p75‐SAP injected into the lateral basal forebrain, the protecting roles of GDNF on pain sensation was abolished (Figure 3). Taken together, these findings provide evidence that GDNF acts by alleviating injury to the cholinergic neurons to reduce the development of chronic postsurgical pain. Nevertheless, further studies are required to precisely modulate GDNF expression or inhibit apoptosis in lateral basal forebrain cholinergic neurons to consolidate this conclusion.

Vierck et al. [25] reported that lesion of the whole basal forebrain cholinergic neurons innervating the cerebral cortex and hippocampus made the rats insensitive to escape from cold and heat stimulation, indicating impaired sensitivity of pain. In contrast, we observed a decrease in mechanical and thermal pain thresholds in mice with selective lesion of the lateral parts of the basal forebrain (Figure 3). The discrepancy suggests that different subregions of the basal forebrain exert distinct modulatory effects on pain processing. This was also supported by earlier reports. Selective destruction of medial septal cholinergic neurons was shown to reduce the pain‐related responses observed in the CA1 region of the dorsal hippocampus following subcutaneous injection of formalin in rats [24], whereas the rats exhibited increased sensitivity to mechanical stimuli following lesion of cholinergic neurons of the NBM area [61]. Furthermore, the extent of NBM injury was found to be closely correlated with the degree of pain sensitivity [61]. The lateral HDB and the MCPO area are also associated with pain, and stimulation of them inhibits pain sensation in cats [62]. These results highlight the importance of precisely modulating the activity of cholinergic neurons of different subregions of the basal forebrain when studying their roles in pain processing.

Several caveats and limitations must be considered when interpreting the findings of this study. First, the selection of only male mice for experiments means that the results and conclusions cannot be extrapolated to both sexes [38, 63]. Second, the mechanism by which perioperative sleep deprivation combined with surgery reduces basal forebrain GDNF concentrations remains unclear. Third, the mice only received 6‐h sleep deprivation, and the sleep duration and structure changes were not measured, which means that it should not be taken for granted that the mice in the sleep deprivation group slept less than the control group throughout the perioperative period.

In conclusion, perioperative sleep deprivation results in decreased GDNF contents and cholinergic neuron activation, and increased cholinergic neuron apoptosis and autophagy dysfunction in the lateral basal forebrain of surgical mice, contributing to increased risk of chronic postsurgical pain.

## Conflicts of Interest

The authors declare no conflicts of interest.

## Supporting information


Appendix S1.

## Data Availability

The data supporting the results are available from the corresponding author upon reasonable request.
