# Supplementary material for: Impaired Basal Forebrain Cholinergic Neuron GDNF Signaling Contributes to Perioperative Sleep Deprivation–Induced Chronicity of Postsurgical Pain in Mice Through Regulating Cholinergic Neuronal Activity, Apoptosis, and Autophagy
Source: CNS Neurosci Ther. 2024 Dec 5;30(12):e70147. doi: 10.1111/cns.70147 (PMC11621383; doi:10.1111/cns.70147)
Supplement: Supplementary file 1 — Appendix S1. [file CNS-30-e70147-s001.zip › cns70147-sup-0001-AppendixS1/cns70147-sup-0001-DataS1.docx]

**
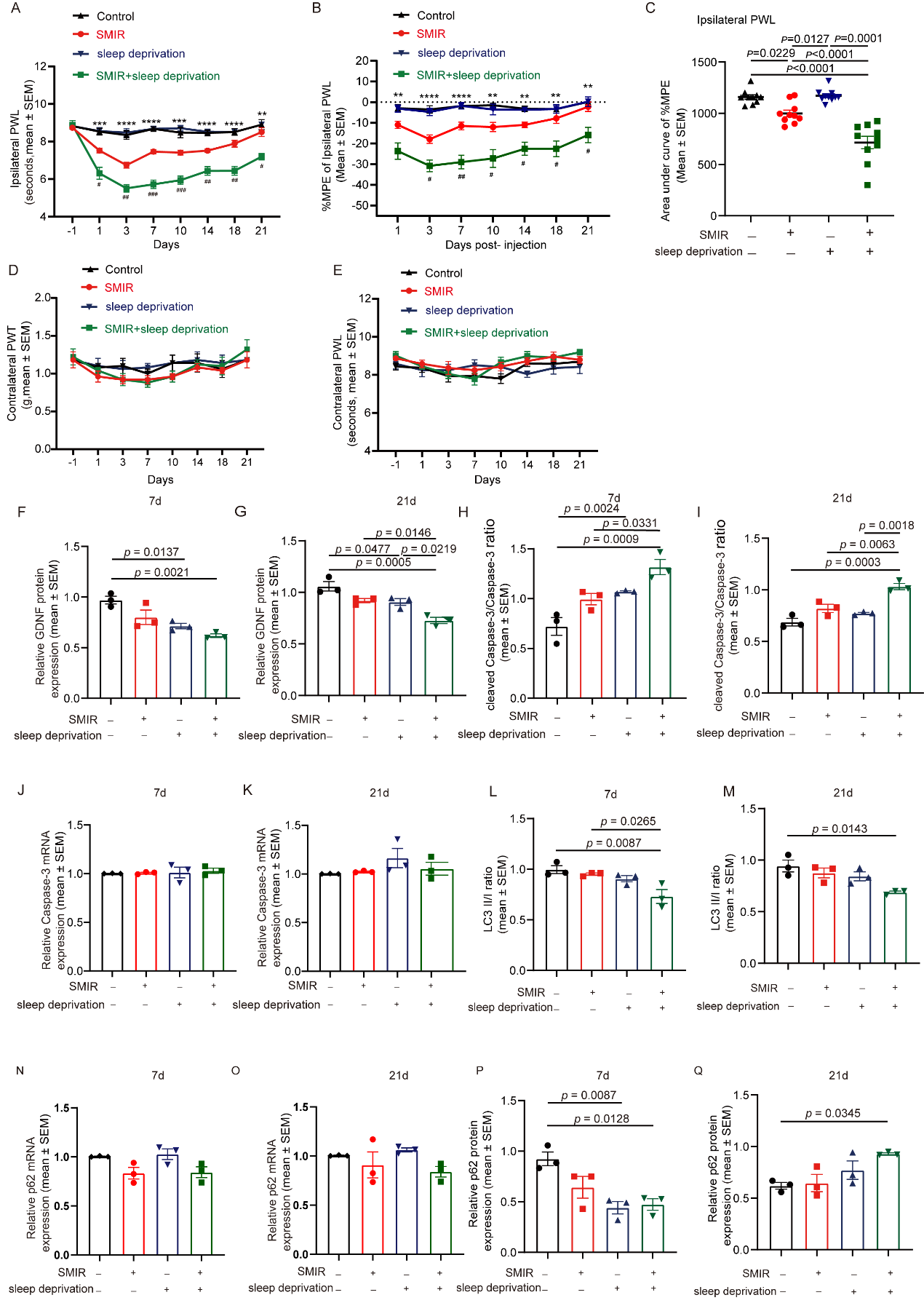
**

**Figure S1. Perioperative sleep deprivation promotes chronicity of postsurgical pain with decreased GDNF contents and increased cholinergic neuronal apoptosis and autophagy dysfunction** **in the basal forebrain.** (**A-E**) Perioperative sleep deprivation was found to significantly increase thermal pain intensity (**A**), decrease the percentage of maximal possible effect (%MPE, **B**), and reduce the area under the curve (AUC) of %MPE (**C**) without affecting contralateral mechanical pain (**D**) and thermal pain (**E**) intensity in SMIR mice. (**F and G**) Expression of GDNF protein at 7 and 21 days after surgery. (**H and I**) Markers of apoptosis (cleaved Caspase-3 and Caspase-3) expression across groups at 7 (**H**) and 21 (**I**) days after surgery. (**J and K**) Expression of Caspase-3 mRNA at 7 (**J**) and 21 (**K**) days after surgery. (**L-M**) Markers of autophagy (LC3 II/I) expression across groups at 7 (**L**) and 21 (**M**) days after surgery. (**N and O**) Expression of p62 mRNA at 7 (**N**) and 21 (**O**) days after surgery. (**P-Q**) p62 protein expression across groups at 7 (**P**) and 21 (**Q**) days after surgery. Ten mice per group were used for the behavioral tests. Biochemical experiments were performed in three independent samples. *, *p* < 0.05; **, *p* < 0.01; ***, *p* < 0.001; and ****, *p* < 0.0001, SMIR + sleep deprivation *vs.* Control. ^#^, *p* < 0.05; ^##^, *p* < 0.01; ^###^, *p* < 0.001; and ^####^, *p* < 0.0001, SMIR + sleep deprivation *vs.* SMIR. Abbreviations: SMIR, skin/muscle incision and retraction; SEM, standard error of the mean.

**
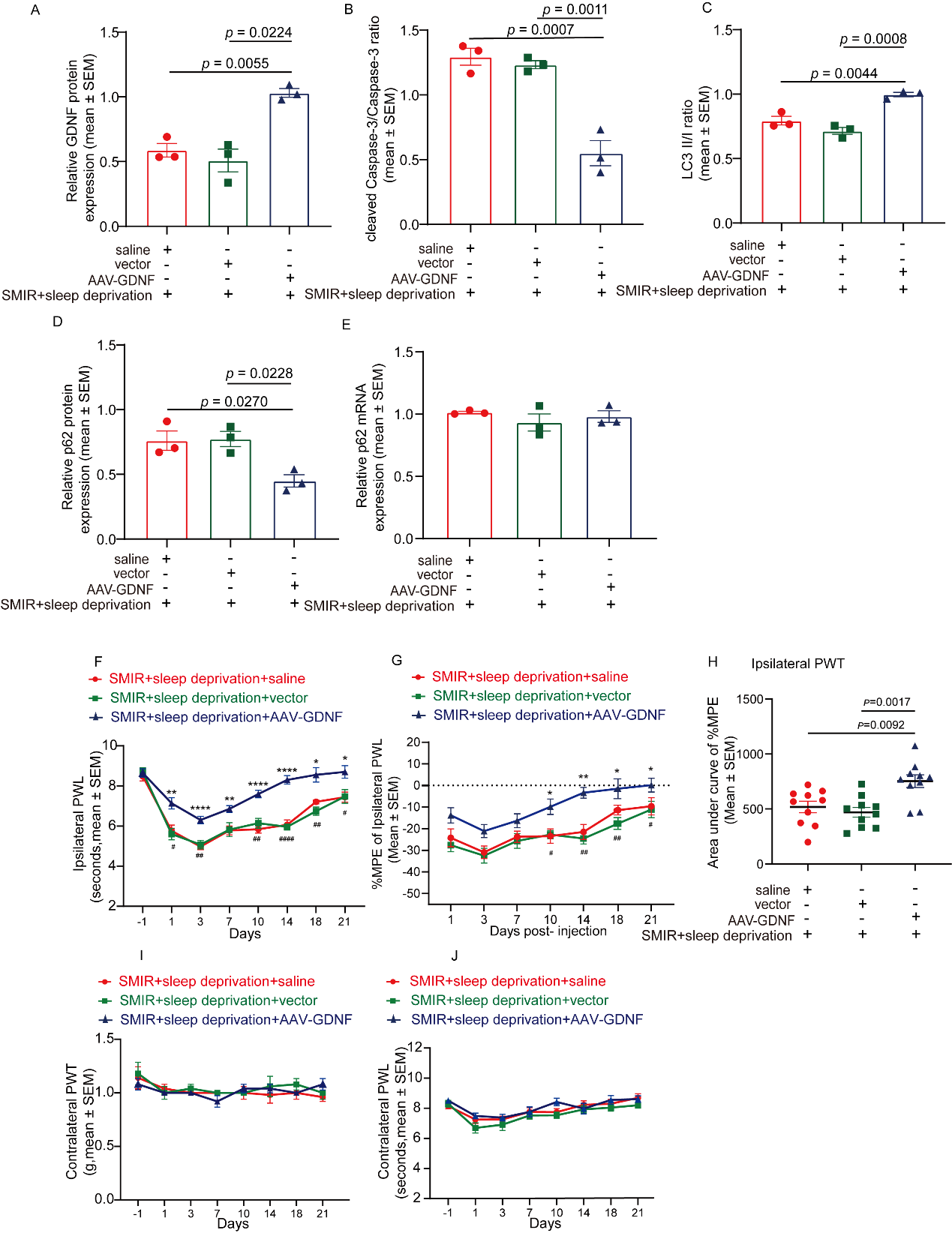
Figure S2. AAV-GDNF promotes GDNF expression, reduces cholinergic neuronal apoptosis and autophagy dysfunction, and counteracts sleep deprivation-induced postoperative chronic pain.** (**A**) Expression of GDNF protein at 21 days after surgery. (**B-E**) Markers of apoptosis (cleaved Caspase-3 and Caspase-3, **B**) and autophagy (LC3 and p62, **C-E**) expression across groups at 21 days after surgery. (**F-J**) AAV-GDNF attenuates the intensity and shortens the duration of thermal pain induced by SMIR combined with sleep deprivation at the ipsilateral hind paws (**F**), increases the MPE (%) (**G**), and increases the AUC (**H**) without affecting mechanical (**I**) and thermal (**J**) pain at the contralateral hind paws in SMIR mice. Ten mice per group were used for the behavioral tests. Biochemical experiments were performed in three independent samples. *, *p* < 0.05; **, *p* < 0.01; ***, *p* < 0.001; and ****, *p* < 0.0001, SMIR + sleep deprivation + saline *vs.* SMIR + sleep deprivation + AAV-GDNF. ^#^, *p* < 0.05; ^##^, *p* < 0.01; ^###^, *p* < 0.001; and ^####^, *p* < 0.0001, SMIR + sleep deprivation + vector *vs.* SMIR + sleep deprivation + AAV-GDNF.


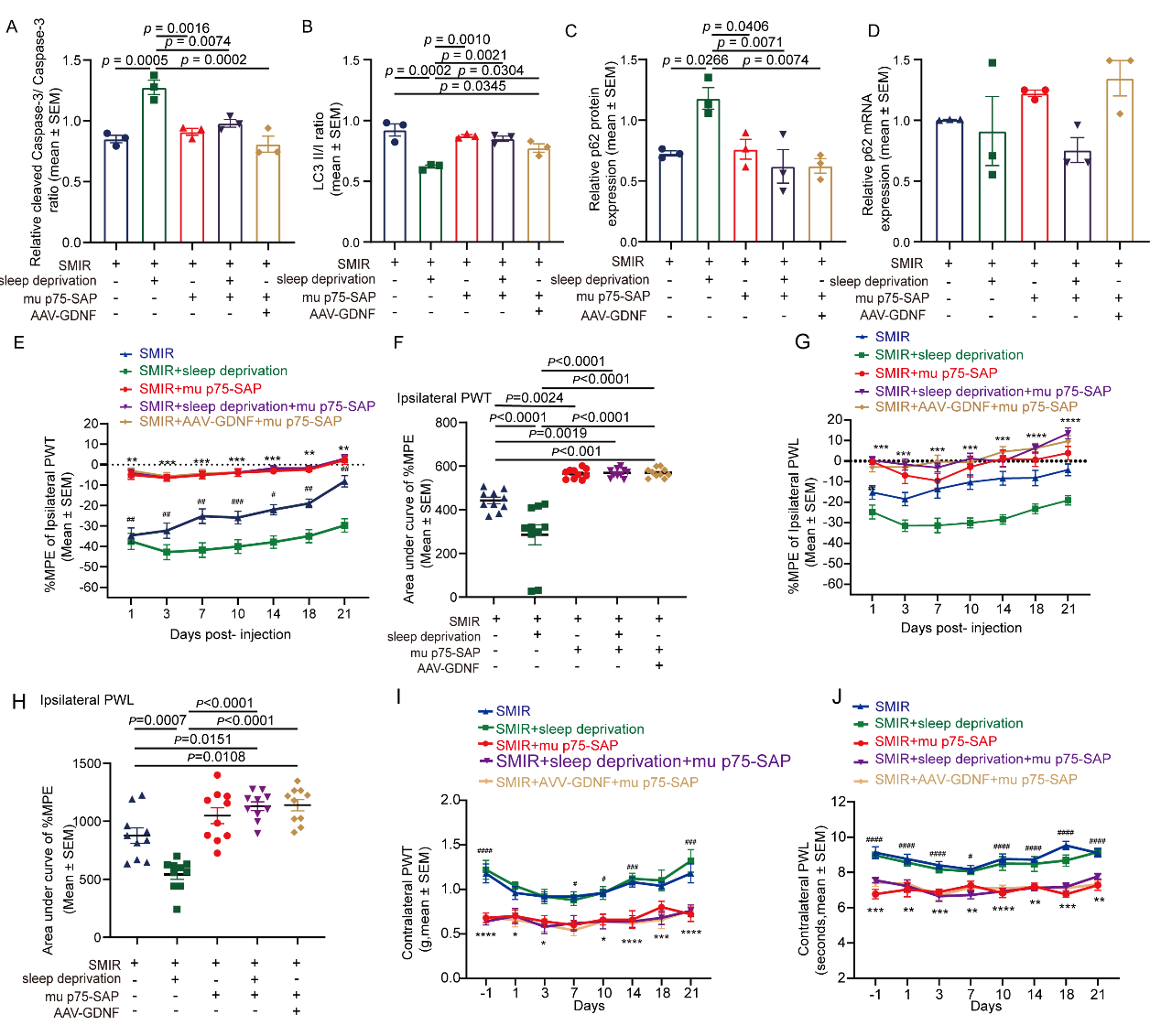


**Figure S3. Mice with lesions of lateral basal forebrain cholinergic neurons are resistant to the pain-enhancing effects of sleep deprivation and the pain-alleviating effects of AAV-GDNF therapy.** (**A-D**) Markers of apoptosis (cleaved Caspase-3 and Caspase-3, **A**) and autophagy (LC3 and p62, **B-D**) expression across groups at 21 days after surgery. (**E-F**) Reduced thermal pain threshold, prolonged mechanical pain duration, increased %MPE of mechanical pain and thermal pain (**E and G**) and AUC (**F and H**) at the contralateral hind paws in mu p75-SAP-treated mice, which were not affected by further sleep deprivation or AAV-GDNF treatment (**I and J**). Ten mice per group were used for the behavioral tests. Biochemical experiments were performed in three independent samples. *, *p* < 0.05; **, *p* < 0.01; ***, *p* < 0.001; and ****, *p* < 0.0001, SMIR + sleep deprivation + mu p75-SAP *vs.* SMIR + sleep deprivation. ^#^, *p* < 0.05; ^##^, *p* < 0.01; ^###^, *p* < 0.001; and ^####^, *p* < 0.0001, SMIR *vs.* SMIR + mu p75-SAP.
