# Supplementary material for: Impaired Basal Forebrain Cholinergic Neuron GDNF Signaling Contributes to Perioperative Sleep Deprivation–Induced Chronicity of Postsurgical Pain in Mice Through Regulating Cholinergic Neuronal Activity, Apoptosis, and Autophagy
Source: CNS Neurosci Ther. 2024 Dec 5;30(12):e70147. doi: 10.1111/cns.70147 (PMC11621383; doi:10.1111/cns.70147)
Supplement: Supplementary file 1 — Appendix S1. [file CNS-30-e70147-s001.zip › cns70147-sup-0001-AppendixS1/cns70147-sup-0002-DataS2.pdf]

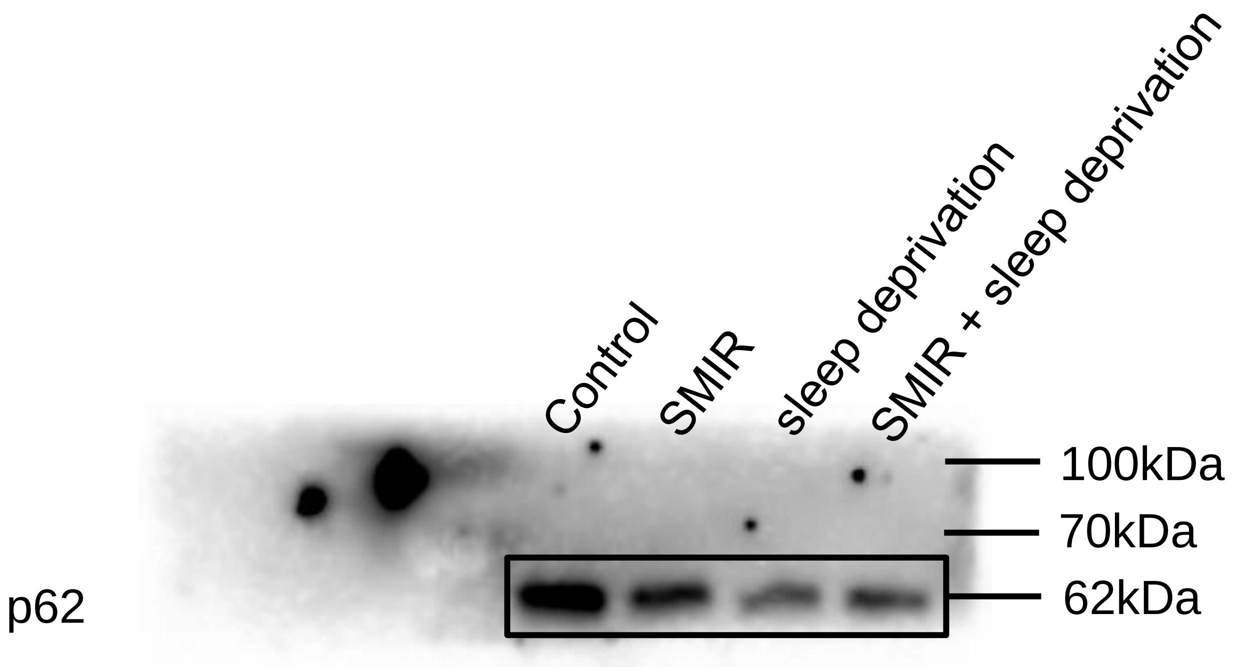

Full unedited gel/blot for Figure 1H

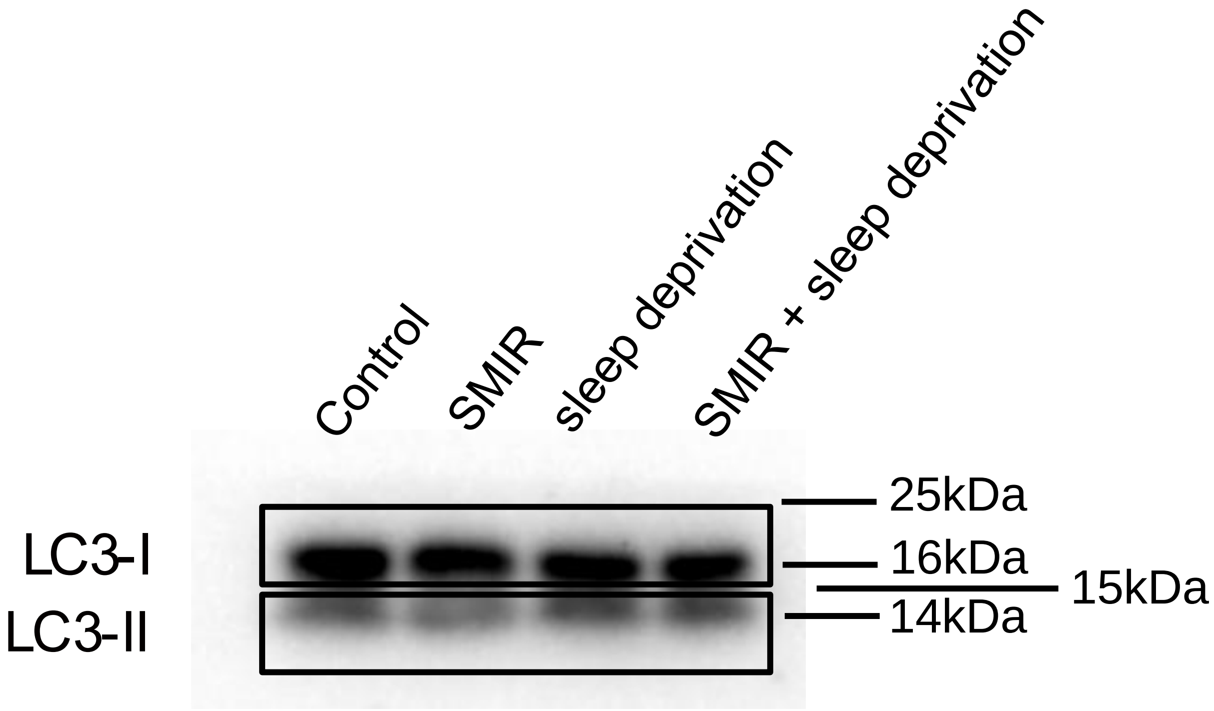

Full unedited gel/blot for Figure 1H

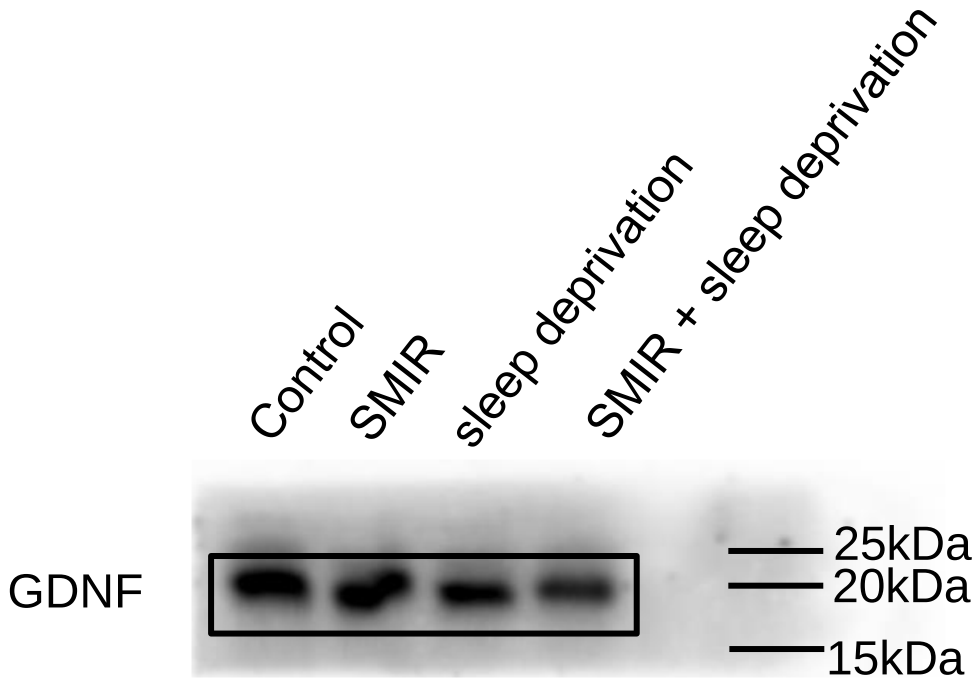

Full unedited gel/blot for Figure 1H

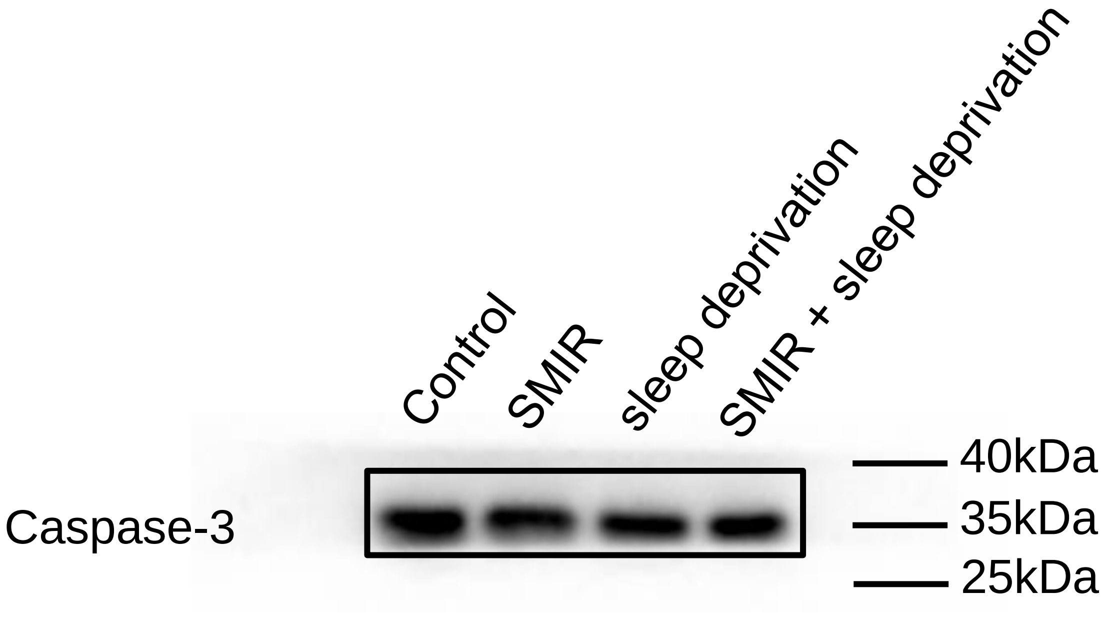

Full unedited gel/blot for Figure 1H

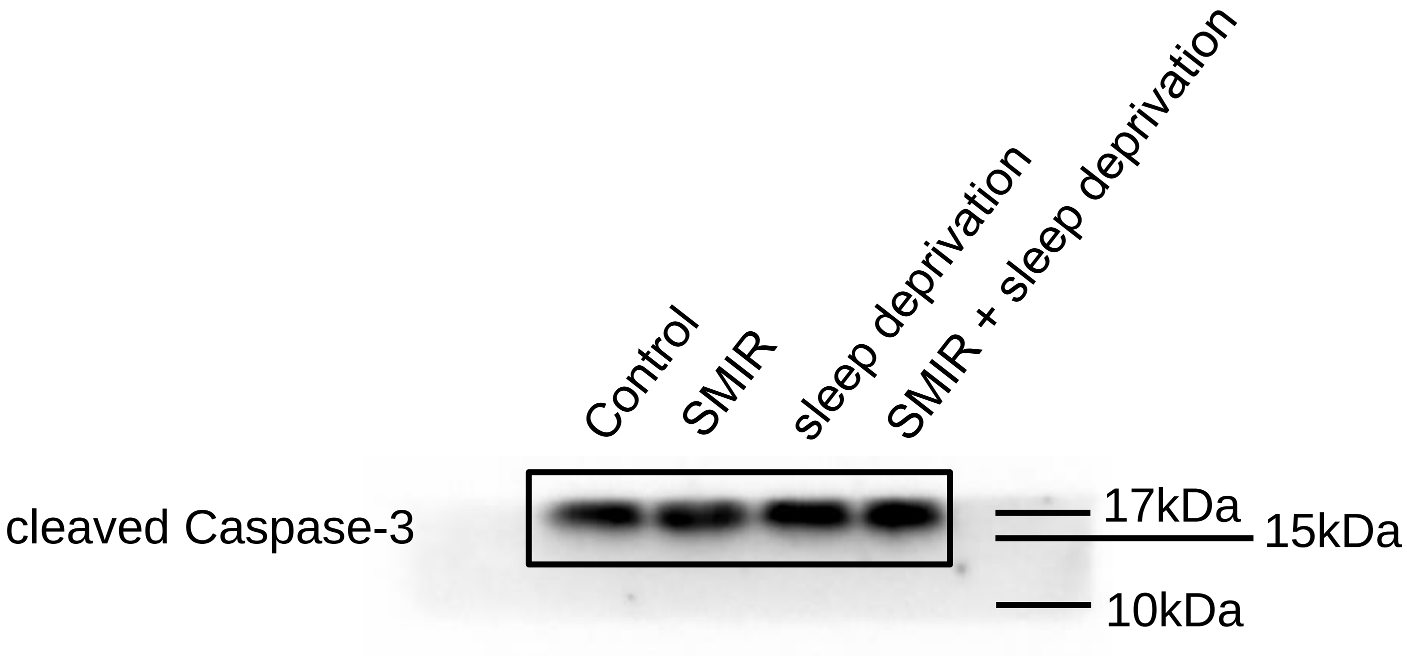

Full unedited gel/blot for Figure 1H

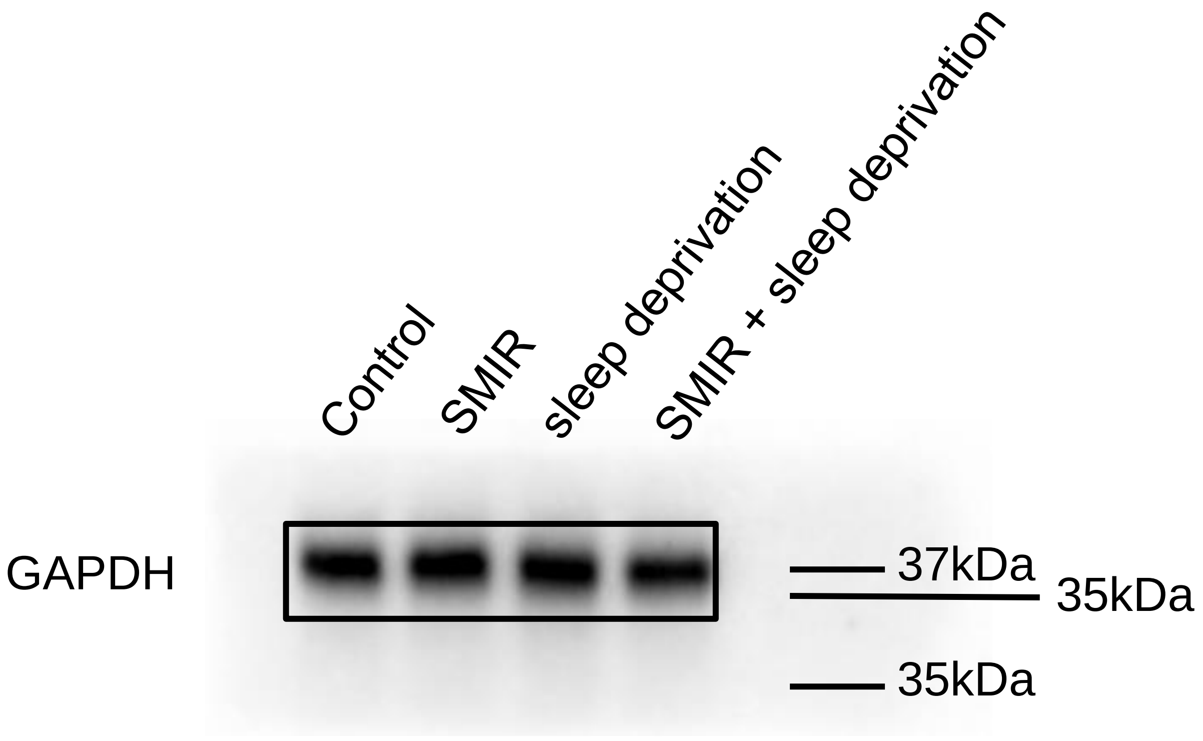

Full unedited gel/blot for Figure 1H

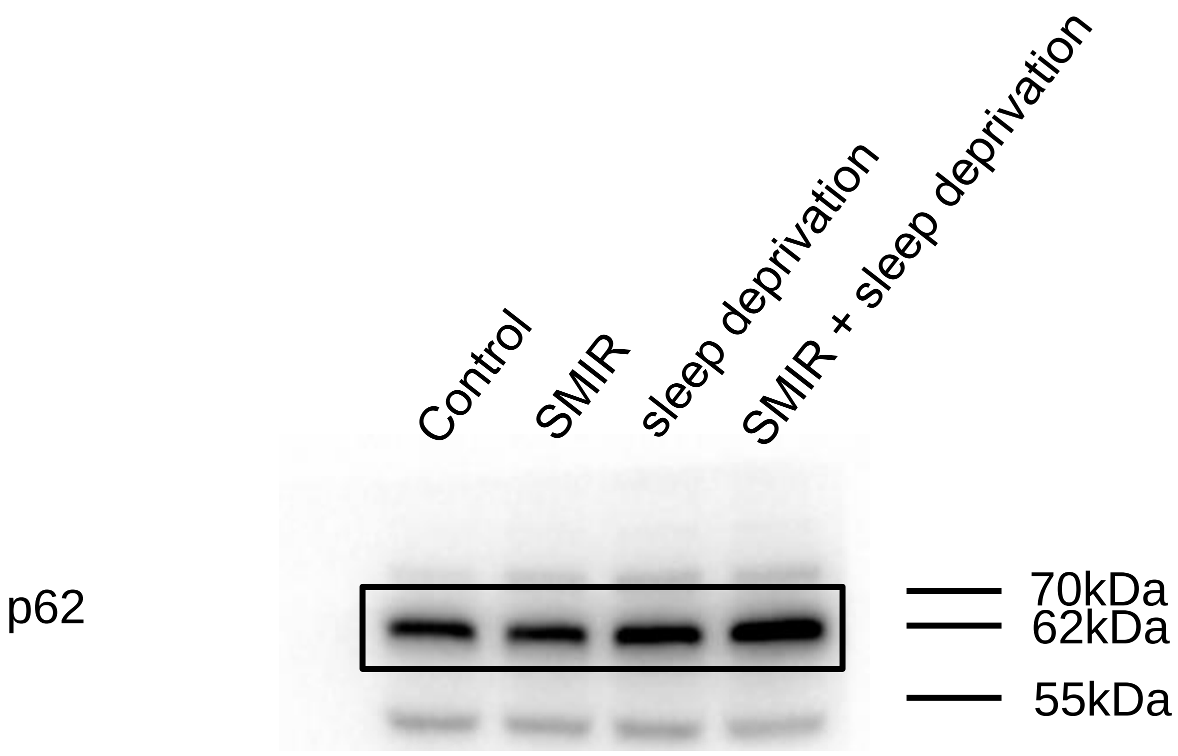

Full unedited gel/blot for Figure 1I

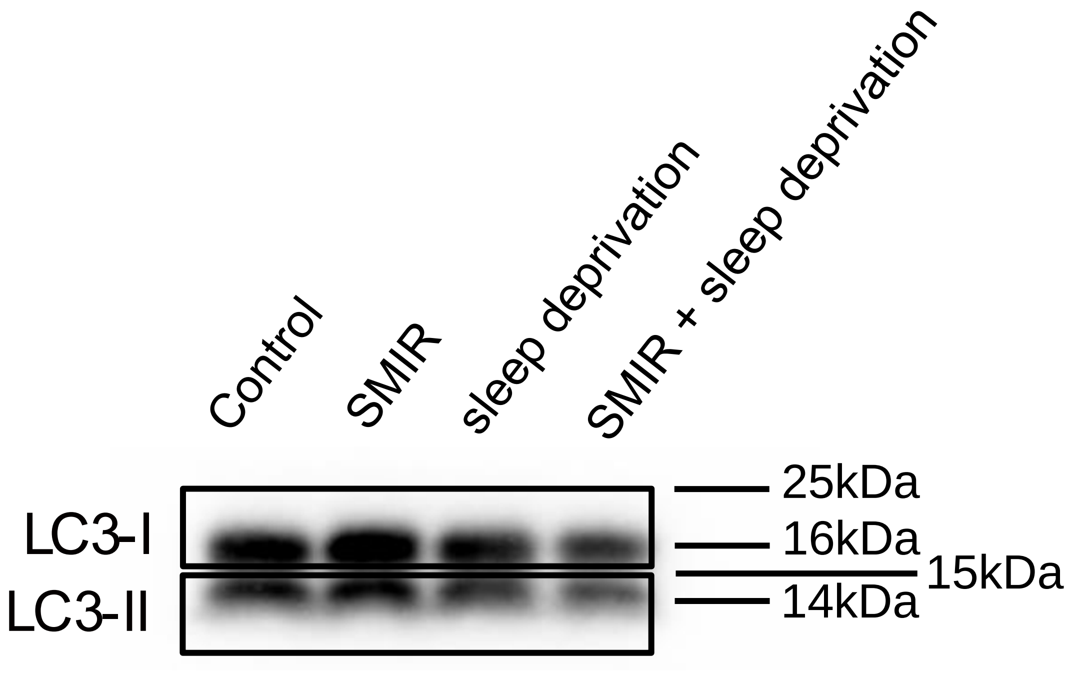

Full unedited gel/blot for Figure 1I

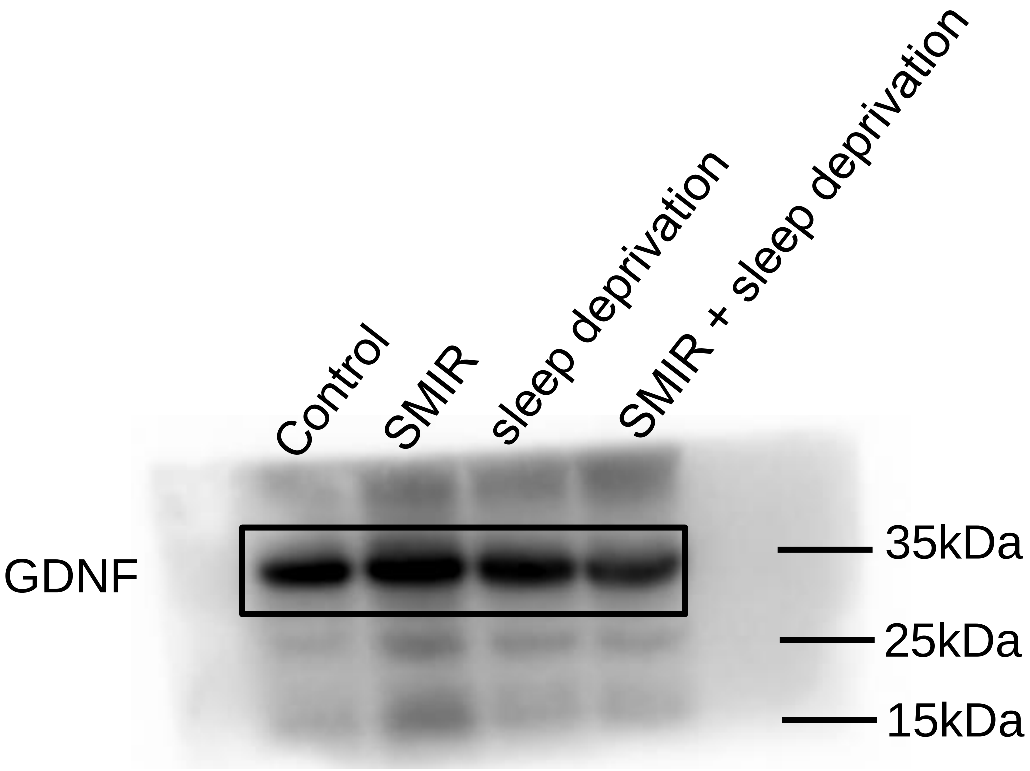

Full unedited gel/blot for Figure 1I

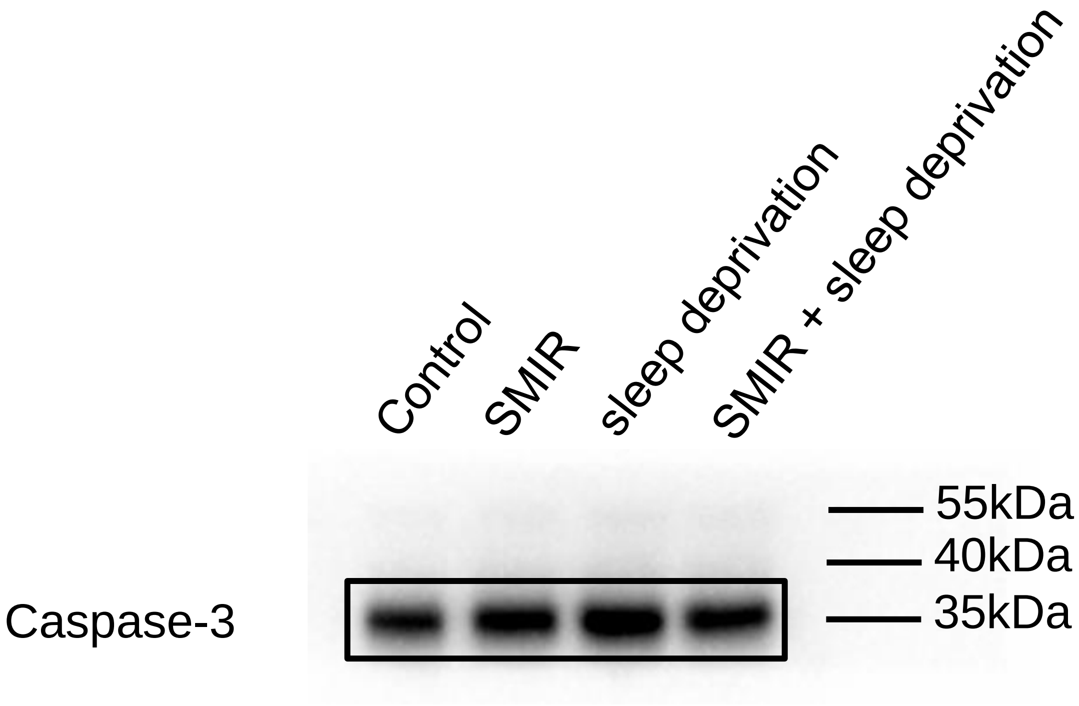

Full unedited gel/blot for Figure 1I

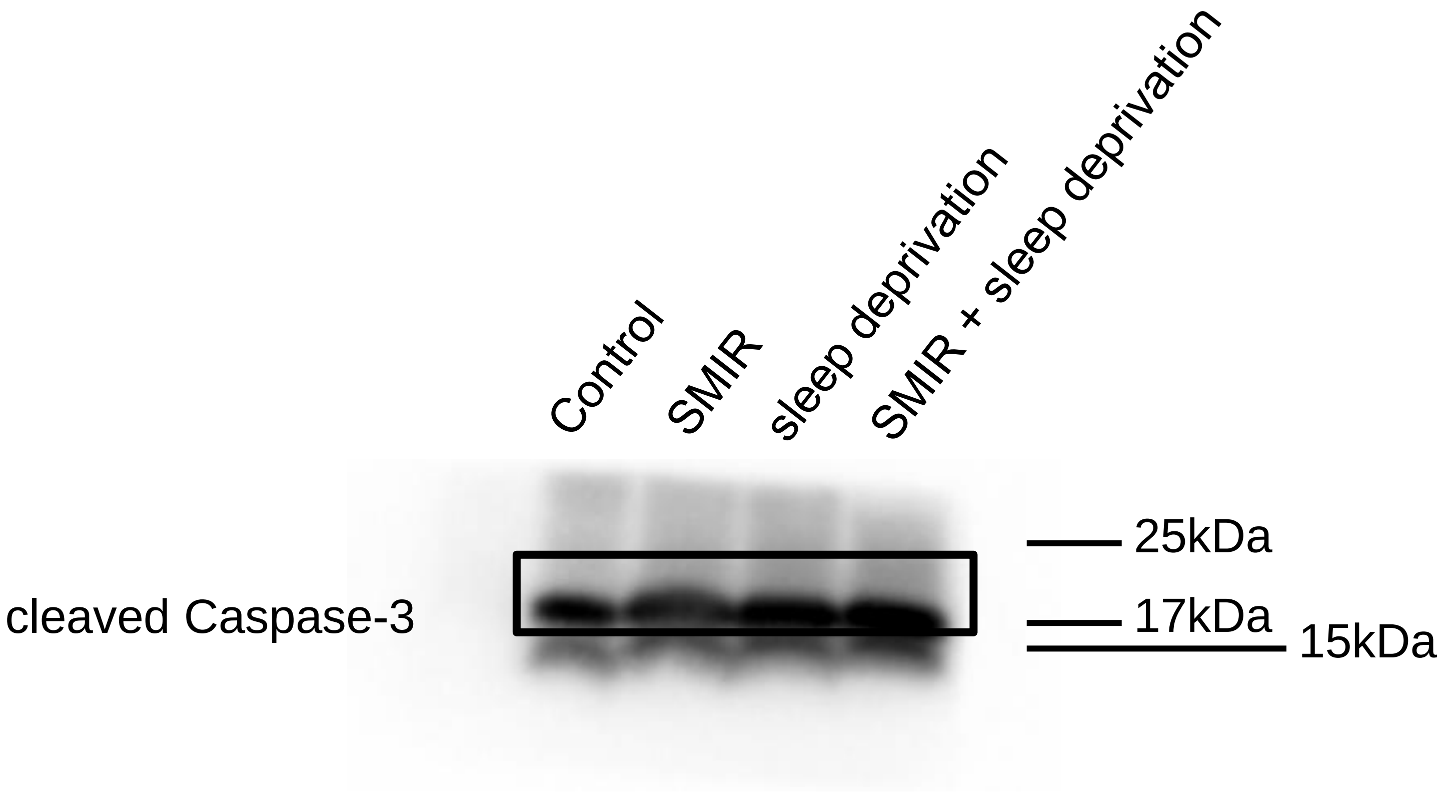

Full unedited gel/blot for Figure 1I

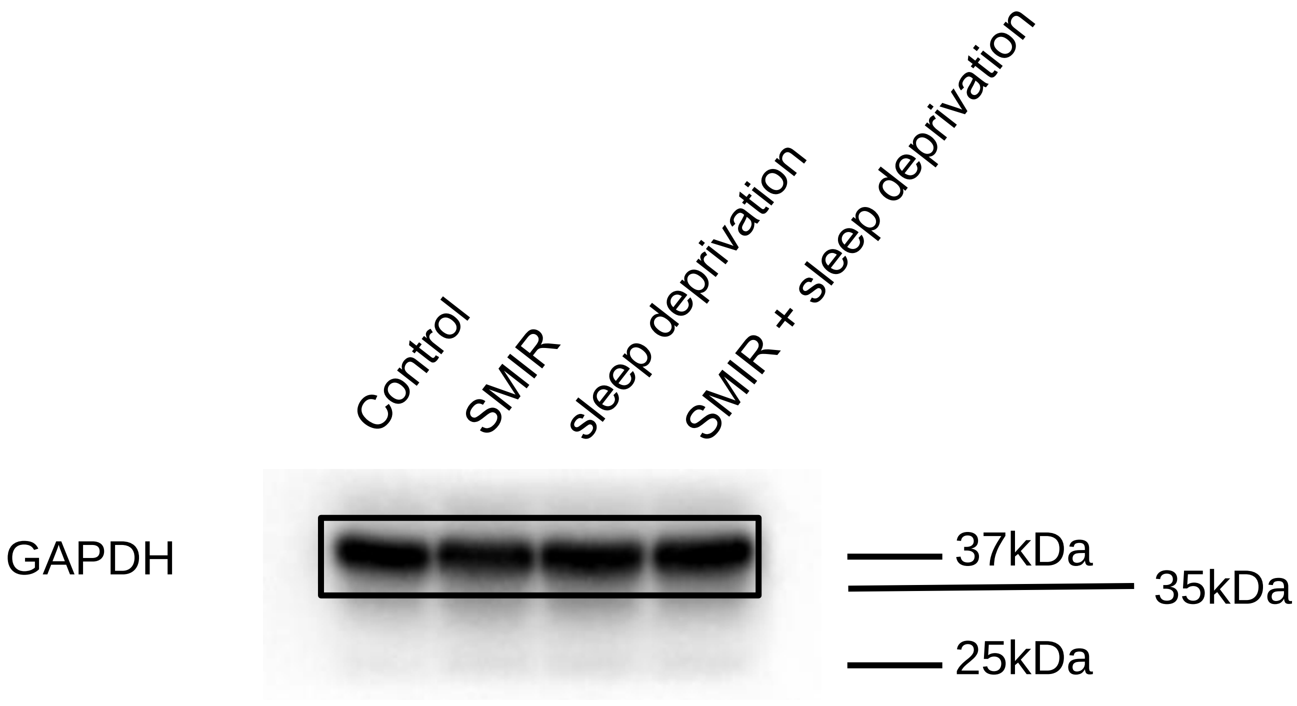

Full unedited gel/blot for Figure 1I

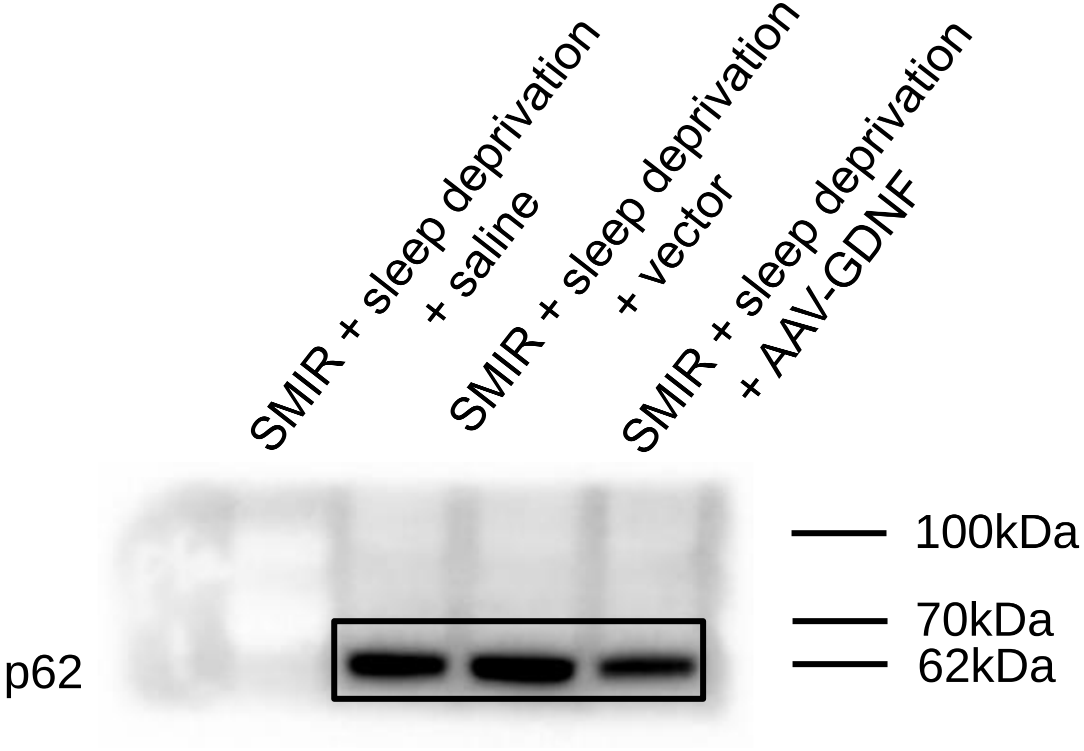

Full unedited gel/blot for Figure 2C

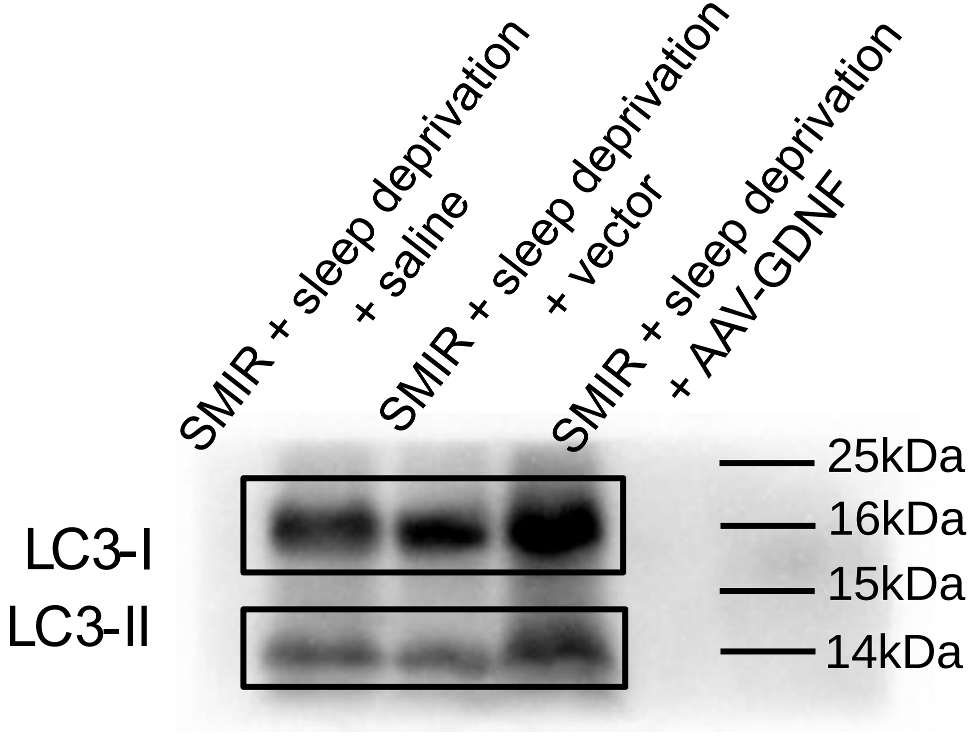

Full unedited gel/blot for Figure 2C

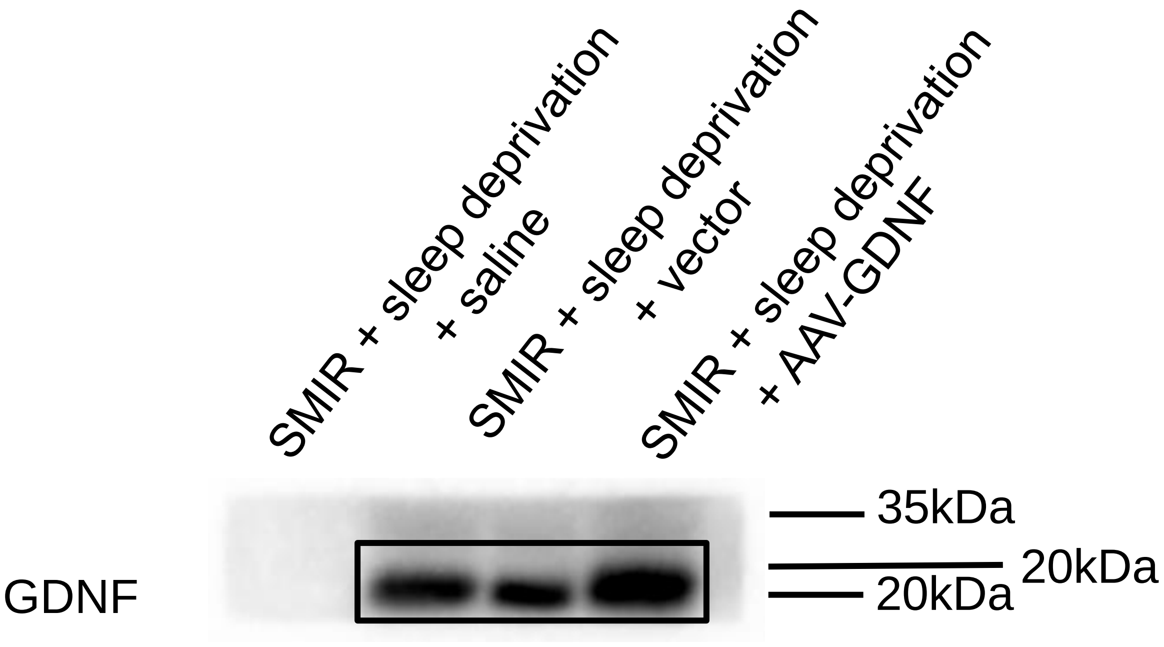

Full unedited gel/blot for Figure 2C

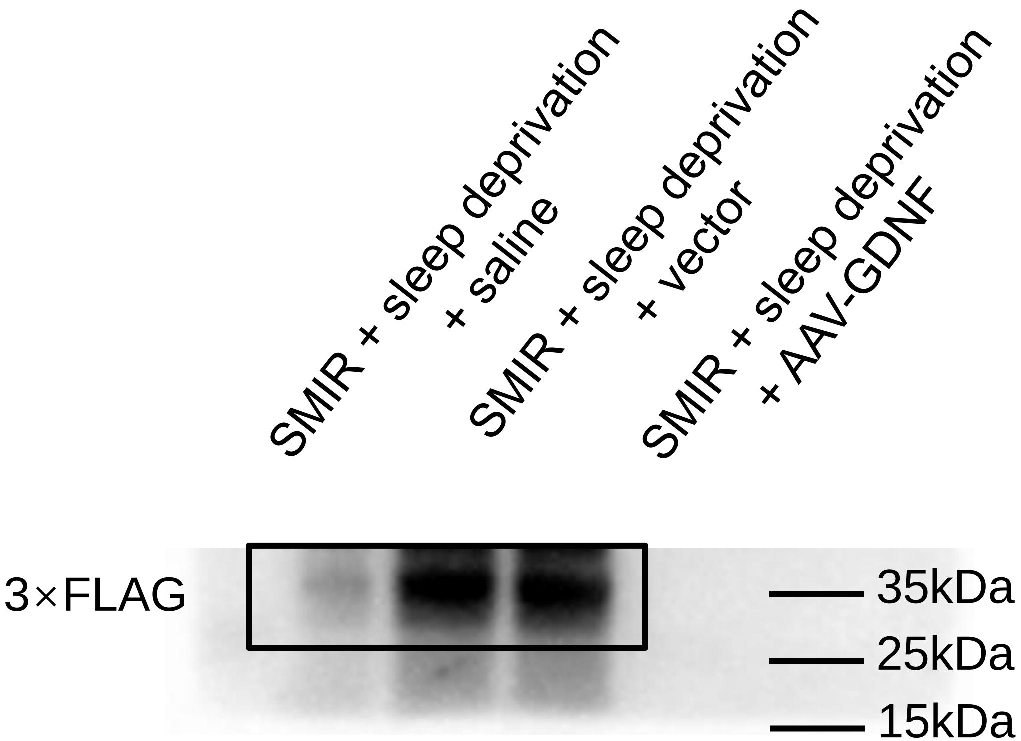

Full unedited gel/blot for Figure 2C

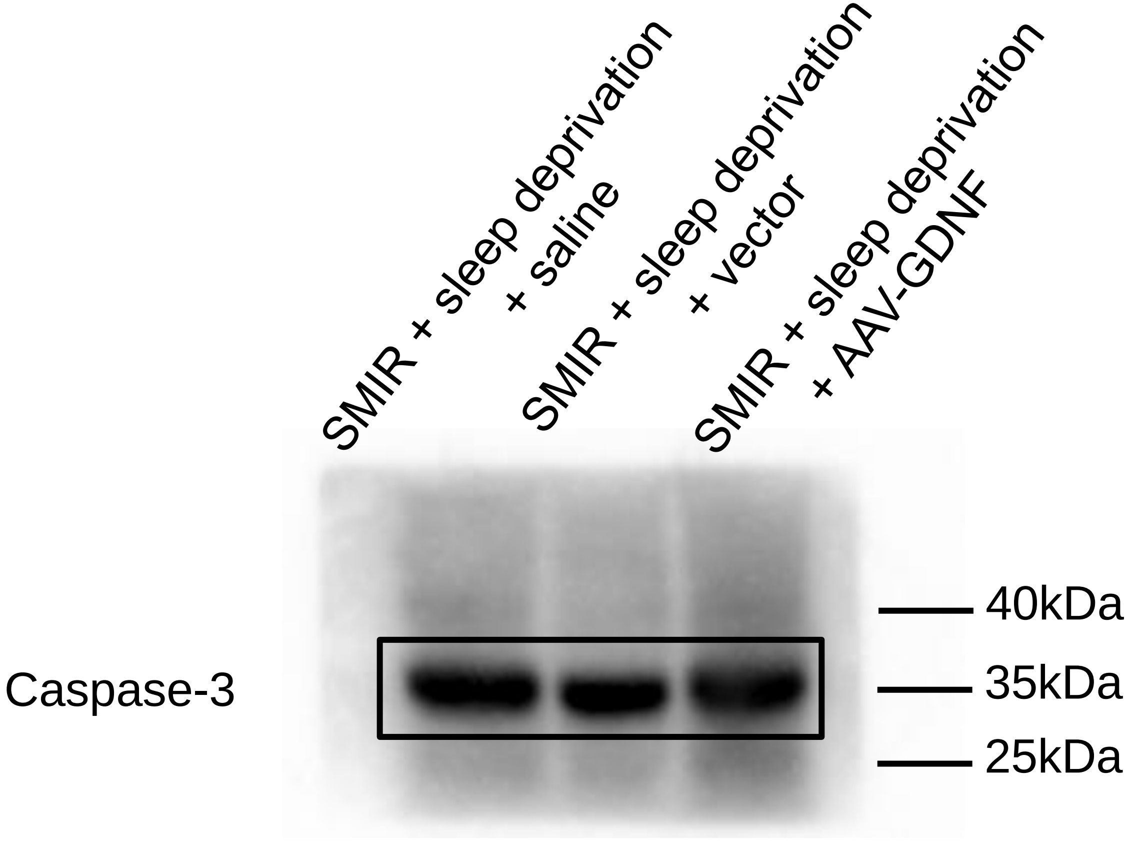

Full unedited gel/blot for Figure 2C

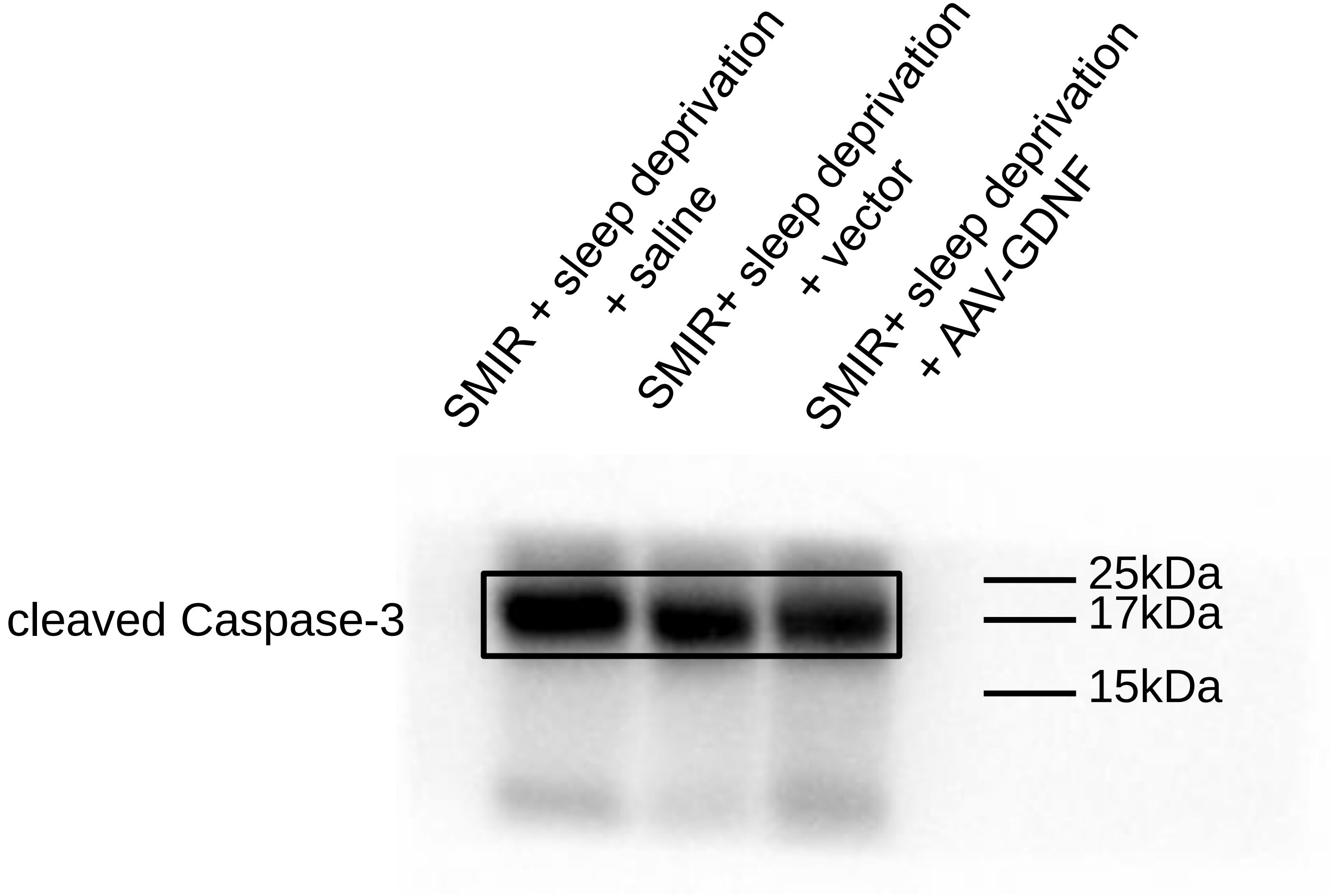

Full unedited gel/blot for Figure 2C

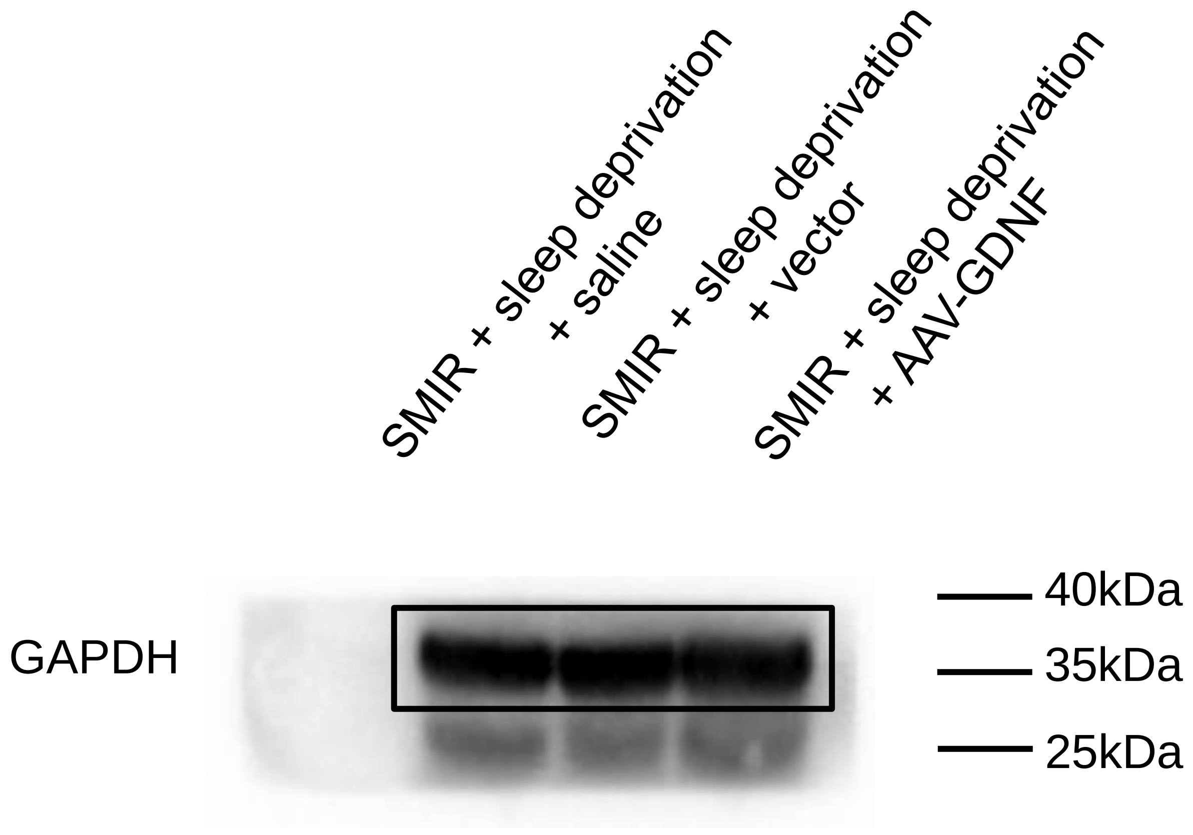

Full unedited gel/blot for Figure 2C

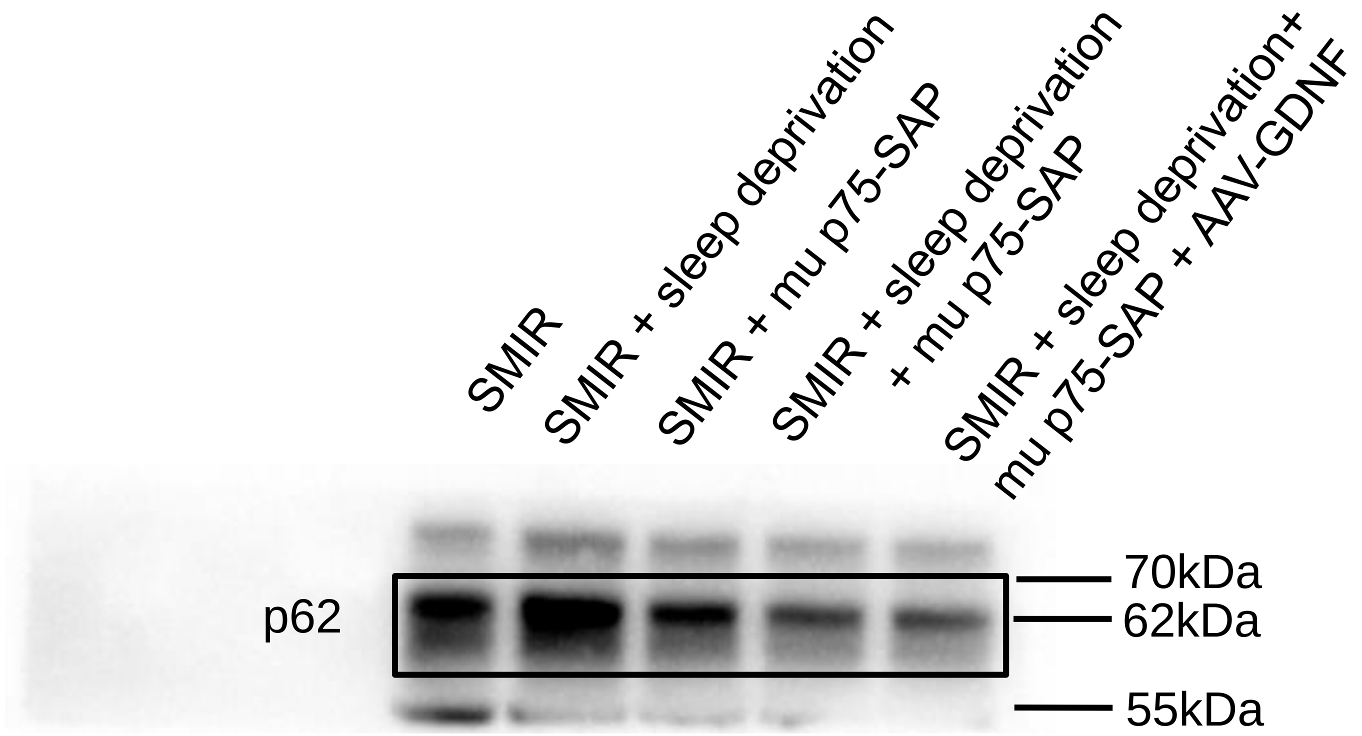

Full unedited gel/blot for Figure 3F

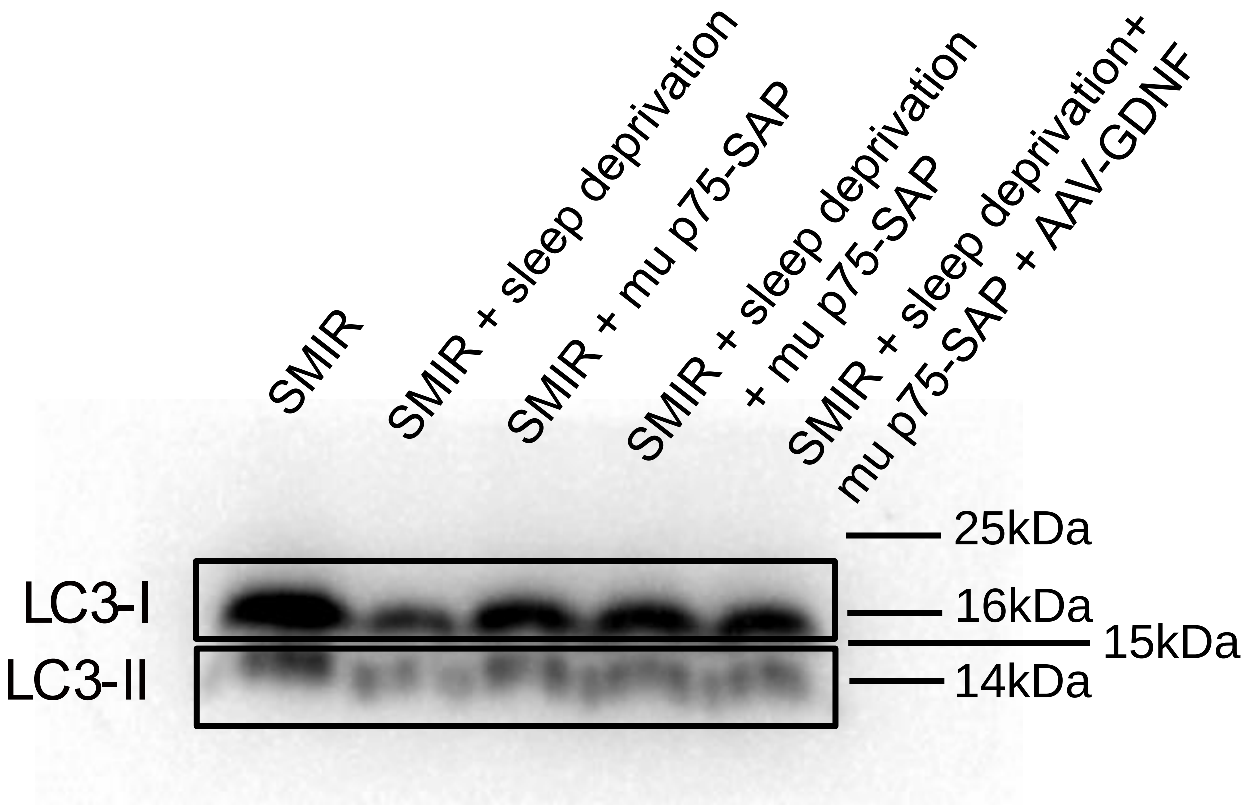

Full unedited gel/blot for Figure 3F

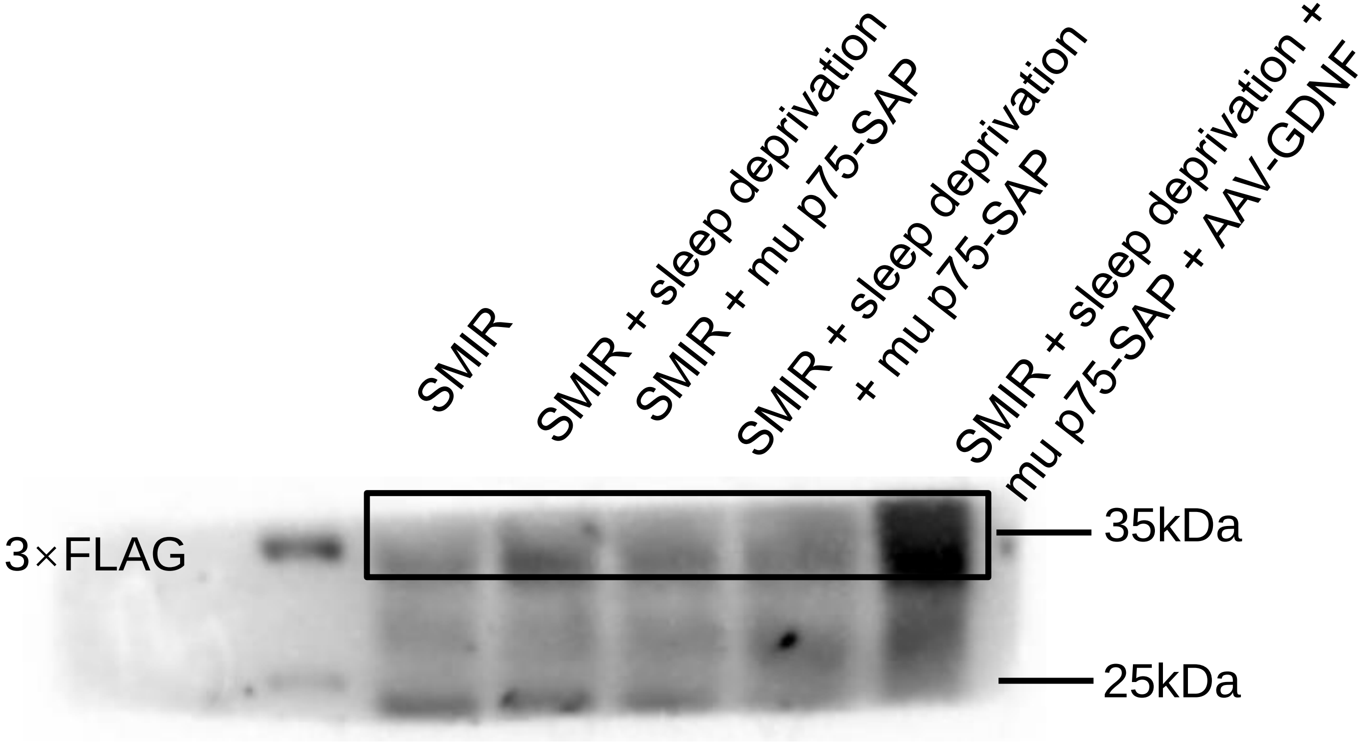

Full unedited gel/blot for Figure 3F

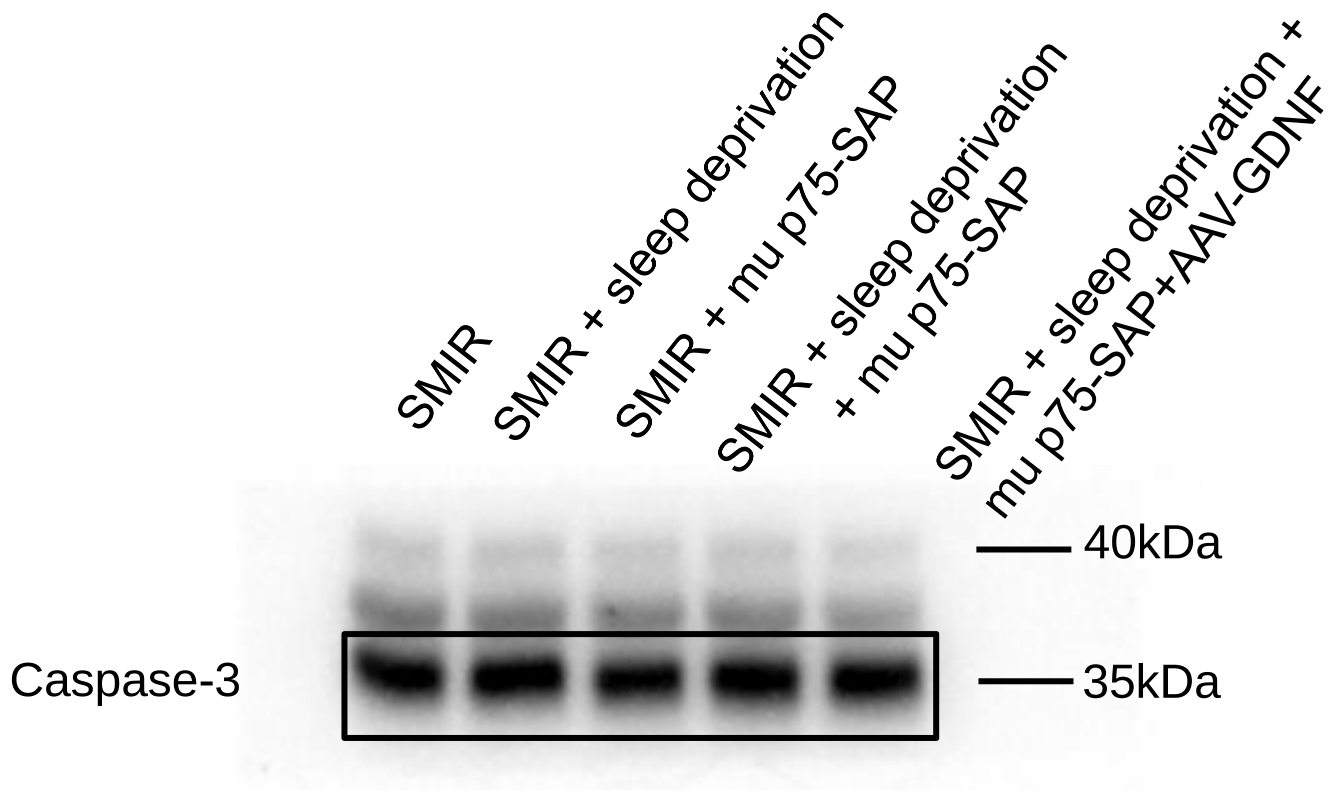

Full unedited gel/blot for Figure 3F

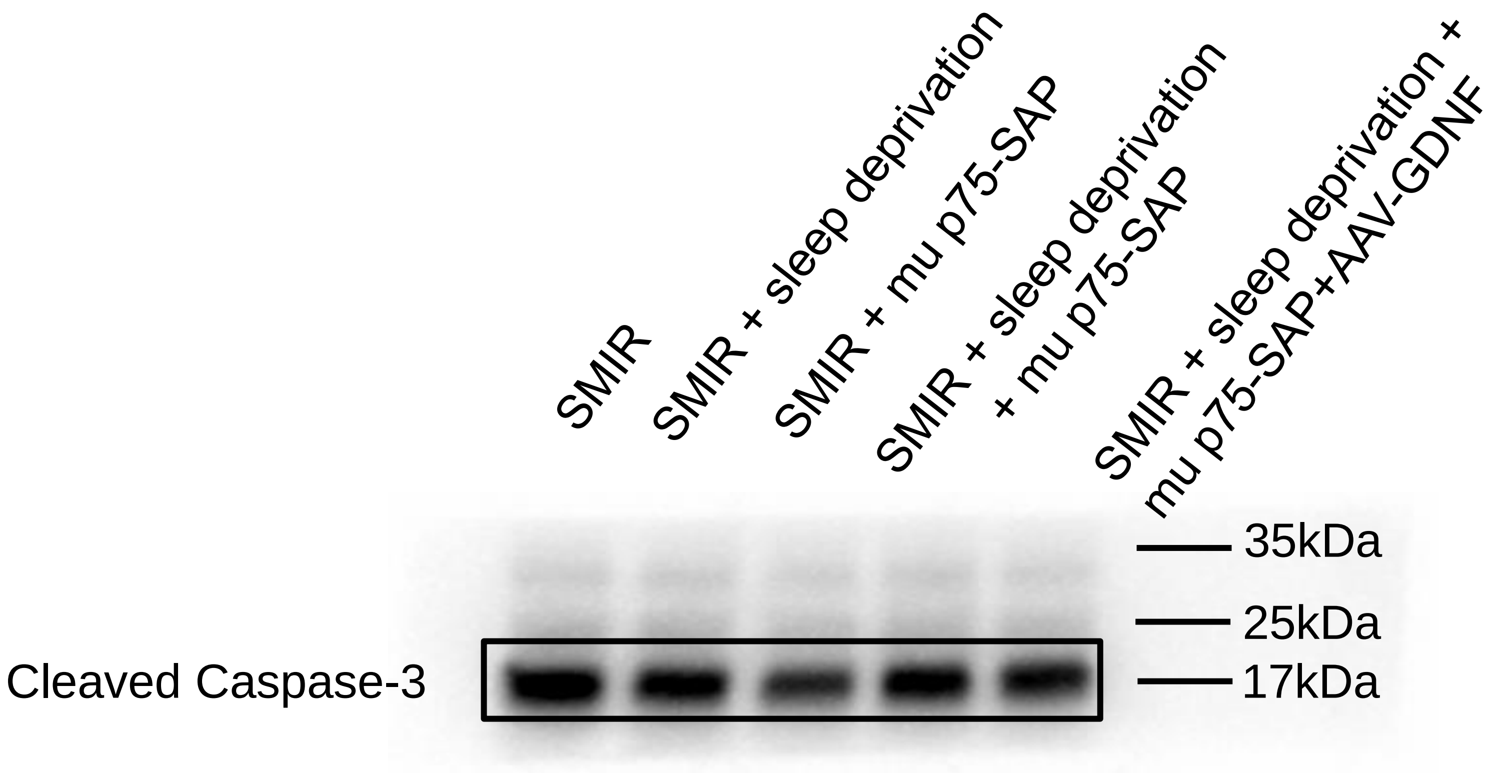

Full unedited gel/blot for Figure 3F

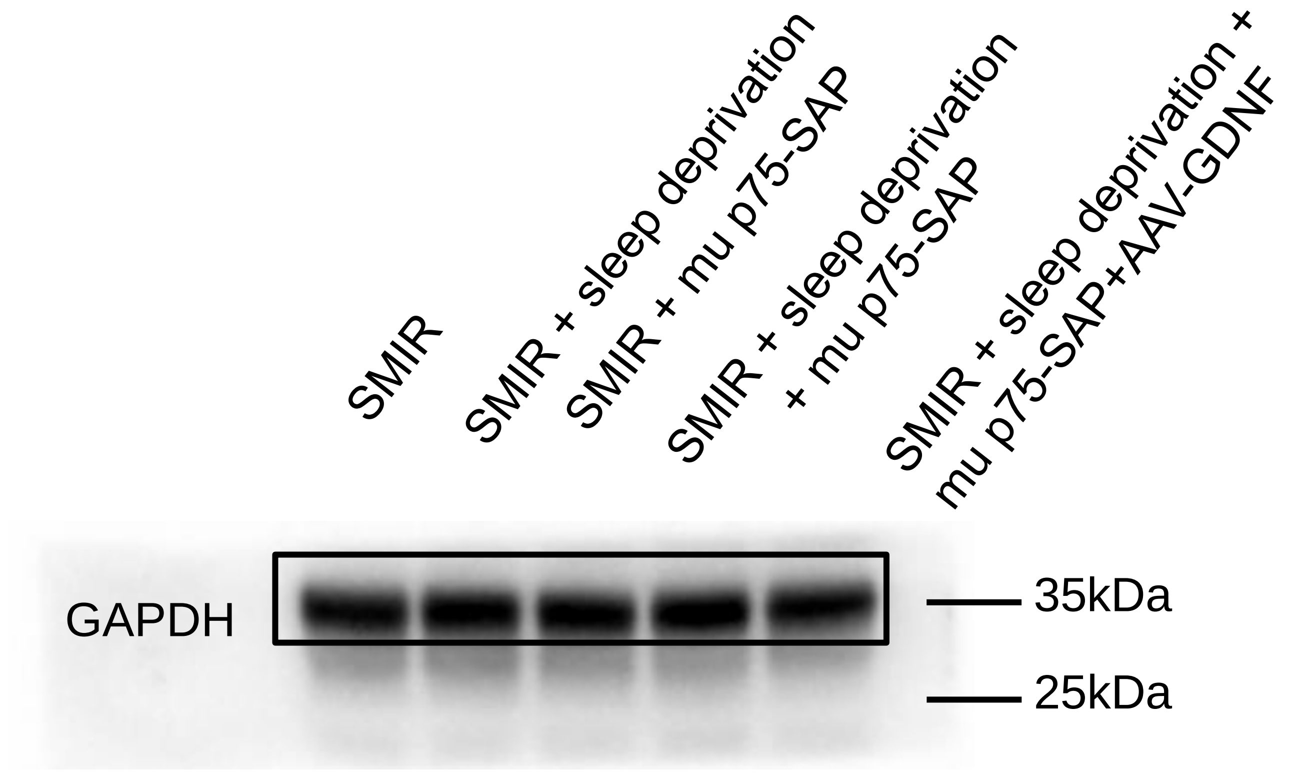

Full unedited gel/blot for Figure 3F
